# Impact of real-time continuous glucose monitoring on glycaemic control in adults with type 2 diabetes: systematic review and meta-analysis

**DOI:** 10.3389/fendo.2025.1761579

**Published:** 2026-01-23

**Authors:** Xia Lian, Ling Jie Cheng, Claire Jun Yi Teo, Isaac Jun Song Tan, Helen Lim, Liang Shen, Jocelyn Han Shi Chew, Wenru Wang, Rinkoo Dalan

**Affiliations:** 1 Department of Endocrinology, Tan Tock Seng Hospital, Singapore, Singapore; 2 Alice Lee Centre for Nursing Studies, Yong Loo Lin School of Medicine, National University of Singapore, Singapore, Singapore; 3 Department of Nursing, Tan Tock Seng Hospital, Singapore, Singapore; 4 National Perinatal Epidemiology Unit, Nuffield Department of Women's and Reproductive Health, University of Oxford, Oxford, United Kingdom; 5 Biostatistics Unit, Yong Loo Lin School of Medicine, National University of Singapore, Singapore, Singapore; 6 Lee Kong Chian School of Medicine, Nanyang Technological University Singapore, Singapore, Singapore

**Keywords:** glycaemic control, real-time continuous glucose monitoring, self-management of diabetes, self-monitoring blood glucose, type 2 diabetes

## Abstract

**Objective:**

To evaluate the effectiveness of real-time continuous glucose monitoring compared with self-monitoring of blood glucose in adults with type 2 diabetes, focusing on glycaemic control, cardiometabolic outcomes, and patient-centred measures.

**Methods:**

Randomised controlled trials published in English with study intervention period ≥12 weeks, which compared real-time continuous glucose monitoring with self-monitoring of blood glucose in adults with type 2 diabetes were included in this systematic review. Analyses were conducted using Review Manager version 9.6. Risk of bias was evaluated using the Cochrane risk-of-bias tool. The Grading of Recommendations Assessment, Development and Evaluations approach was used to assess certainty of evidence.

**Data Sources:**

The search was conducted across PubMed, CINAHL, Web of Science, the Cochrane Library databases and ClinicalTrials.gov from inception to July 2025.

**Results:**

This systematic review was reported in accordance with the Preferred Reporting Items for Systematic Reviews and Meta-Analyses guidelines. Eleven studies which compared real-time continuous glucose monitoring (n=437) with self-monitoring of blood glucose (n=352) were included. Real-time continuous glucose monitoring use was associated with a significant reduction in HbA1c (mean difference=−0.20%), improved time-in-range (mean difference=7.41%), reduced time-above-range (mean difference=6.93%) and reduced time-below-range (mean difference=0.26%). Glucose variability was significantly lower (mean difference=-1.06%) and users demonstrated greater improvements in readiness for diabetes self-management (standardised mean difference=0.69). No significant differences were observed in cardiometabolic or psychosocial outcomes.

**Conclusion:**

Real-time continuous glucose monitoring improves glycaemic control and self-management capacity compared with self-monitoring of blood glucose in adults with type 2 diabetes. These findings support the integration of real-time continuous glucose monitoring into routine clinical care, particularly for individuals requiring intensive glucose monitoring and tailored self-care support.

**Systematic review registration:**

https://www.crd.york.ac.uk/prospero/, identifier CRD42025625444.

## 1 Introduction

Type 2 diabetes represents a growing global health challenge, with affected individuals facing significantly higher cardiovascular disease risk compared to the general population (1, 2). Recent evidence emphasises the clinical significance of glucose metrics - time in range (TIR), time above range (TAR), time below range (TBR) and glucose variability - as crucial indicators of cardiovascular outcomes and mortality (3, 4). Effective management of these metrics, together with cardiovascular risk factors including body weight, body mass index (BMI), lipid profile, and blood pressure (BP), plays a vital role in preventing adverse health outcomes (5, 6).

The traditional method of self-monitoring blood glucose (SMBG) has several limitations. Firstly, it necessitates frequent finger-prick testing, which is both painful and inconvenient, often leading to poor concordance among individuals with type 2 diabetes. Additionally, the intermittent nature of these measurements frequently misses important glucose fluctuations, potentially delaying treatment adjustments (7). While glycated haemoglobin (HbA1c) serves as a validated biomarker for long-term glycaemic control and complication risk (8, 9), it only reflects average glucose levels over 2–3 months, without capturing daily glucose patterns or hypoglycaemic events (8, 9).

Continuous glucose monitoring (CGM) overcomes these limitations by delivering real-time glucose measurements from interstitial fluid every 1 to 5 minutes, providing comprehensive insights into glucose patterns and trends that enable more timely treatment adjustments (10). There are three main types of CGM: professional CGM, which is used in clinical settings for temporary glucose monitoring; intermittently scanned CGM (isCGM), which requires users to scan the sensor to obtain glucose readings; and real-time CGM (rtCGM), which automatically transmits glucose data. Among these, rtCGM has demonstrated significant advantages in facilitating timely glucose self-monitoring through automatic data transmission and immediate alerts for glucose excursions (11, 12). This technology enhances users’ awareness of glucose patterns and provides instant feedback on lifestyle modifications (13). Furthermore, its automation has been shown to achieve better glycaemic control compared to retrospective CGM (14, 15).

While CGM has demonstrated clear benefits in individuals with type 1 diabetes (15–17), its role in type 2 diabetes management continues to evolve (18, 19). Previous systematic reviews have taken a broader approach by examining various types of CGM, with a primary focus on HbA1c outcomes (20–24). This review, however, specifically focuses on evaluating the comprehensive impact of rtCGM in adults with type 2 diabetes, irrespective of insulin use, and explores a wider range of associated outcomes.

### 1.1 Aims

The primary objective of this review is to assess the effectiveness of rtCGM compared to SMBG in improving glycaemic control in adults with type 2 diabetes. The secondary objectives include examining the effects of rtCGM on glucose metrics, cardiometabolic parameters, self-care behaviours, quality of life, diabetes treatment satisfaction, and adverse events.

## 2 Methodology

### 2.1 Study design

The methodology of this systematic review adhered to the Cochrane Handbook for Systematic Reviews of Interventions (25), whilst the reporting framework followed Preferred Reporting Items for Systematic Reviews and Meta-Analyses (PRISMA) guidelines. The PRISMA checklist is provided in **Supplementary Data 1**. This review was registered on the International Prospective Register of Systematic Reviews (PROSPERO) with registration number CRD42025625444.

### 2.2 Data sources and searches

We initially searched PROSPERO and the Cochrane Database of Systematic Reviews to identify existing or ongoing reviews to avoid duplication. A comprehensive search was conducted across four electronic databases: PubMed, CINAHL, Web of Science, and the Cochrane Library. The search initially covered all publications from database inception to 24 September 2024 and was subsequently updated in July 2025. No restrictions were applied regarding language or publication date; however, only articles published in English were reviewed. To identify unpublished and ongoing studies, we searched clinical trial registries (https://clinicaltrials.gov). Reference lists of included studies and relevant systematic reviews were manually screened.

A comprehensive search strategy was developed for PubMed in collaboration with the university librarian and was subsequently adapted for use in the other databases. The search terms combined medical subject headings (MeSH) and keywords related to population, intervention, and study design, then searches with title and abstract fields (**Supplementary Data 2**). Cochrane’s highly sensitive search strategies were employed to optimise randomised controlled trial identification (26). Reference management and duplicate removal were performed using EndNote X20 software (27).

### 2.3 Inclusion and exclusion criteria

Studies were included if they met the following eligibility criteria: adults aged 18 years and older with type 2 diabetes, comparison of rtCGM with SMBG, and reporting of HbA1c as an outcome measure. To ensure high-quality evidence and consistency in data interpretation, this review included only randomised controlled trials published in English with a minimum intervention duration of 12 weeks, corresponding to the measurement period reflected by HbA1c. Studies were excluded if they involved pregnant women or individuals with type 1 diabetes. Additionally, studies utilising isCGM or professional CGM were excluded, as these modalities do not offer predictive alerts for glucose fluctuations.

Two reviewers (LX, TJY) independently conducted the selection process using predefined criteria. Initial screening of titles and abstracts was performed using Rayyan software (Rayyan Systems Inc, Cambridge), preceded by a pilot screening of twenty records to ensure consistent application of selection criteria. Studies were marked as ‘Maybe’ if potentially relevant or unclear, while exclusions were documented with reasons. Full-text assessment of eligible studies was then conducted independently by both reviewers. Disagreements were resolved through discussion, and with a third reviewer (WW) if necessary. Two primary study authors were contacted for clarification; but no responses were received, hence these studies were excluded. The selection process was documented using a PRISMA flowchart to ensure transparent reporting across all phases.

### 2.4 Data extraction and quality assessment

Data extraction was performed using a standardised form adapted from the Cochrane Handbook guidelines (25). The form captured key study characteristics including author’s name, publication year, participant demographics, number of participants, mean age of participants, diabetes treatment, sensor usage pattern, intervention duration, intervention/comparator, and outcome variables. Two reviewers (LX and TJY) initially piloted the extraction form on twenty studies to ensure consistency. Inter-rater reliability was assessed using Kappa statistics (28), yielding a coefficient of 0.81, indicating strong agreement between reviewers. They subsequently extracted data independently from all included studies. Discrepancies were resolved through discussion, with study authors contacted for clarification where necessary. For studies with multiple publications, data were initially extracted separately and later consolidated. One reviewer (LX) entered the data into Review Manager Web (29), while the second reviewer (TJY) independently verified all entries for accuracy.

### 2.5 Outcomes

The primary outcome was defined as the change in HbA1c levels from baseline to study completion. Secondary outcomes encompassed four key domains. First, glucose metrics were assessed through TIR (percentage of time with glucose 3.9–10 mmol/L), TAR (percentage of time with glucose >10 mmol/L), TBR (percentage of time with glucose <3.9 mmol/L) and glucose variability (coefficient variation %). Second, cardiometabolic parameters included anthropometric measures (body weight in kg and BMI in kg/m²), lipid profile [low density lipoprotein (LDL), high density lipoprotein (HDL), and triglycerides in mmol/L], and BP measurements [systolic (SBP) and diastolic (DBP) in mmHg]. Third, self-reported outcomes comprised self-care behaviour, quality of life, diabetes treatment satisfaction and adverse events.

### 2.6 Risk of bias and certainty of evidence assessment

Risk of bias was assessed using the Cochrane Risk-of-Bias tool (RoB 2.0) (25). Two independent reviewers (LX and TJY) evaluated five domains: [1] bias arising from the randomisation process, [2] bias due to deviations from intended interventions, [3] bias due to missing outcome data, [4] bias in outcome measurement, and [5] bias in the selection of the reported result. Each study was classified as having a low risk of bias, some concerns for bias, or a high risk of bias. Trials were labelled as low risk of bias only if all five domains were rated as low risk. Disagreements were resolved through discussion. The risk-of-bias assessments were visualised using Review Manager (RevMan) Web (29).

The certainty of evidence was assessed using the Grading of Recommendations Assessment, Development and Evaluations (GRADE) framework through the GRADEpro tool (https://gdt.gradepro.org) (30) and categorised as high, moderate, low, and very low quality. Any disagreements between reviewers were resolved through discussion.

### 2.7 Data synthesis and analysis

A meta-analysis was conducted using Review Manager (RevMan) version 9.6 (The Cochrane Collaboration, Copenhagen), with a p-value of <0.05 considered statistically significant (31). Effect sizes were calculated as mean difference (MD) with 95% confidence interval (CI) for continuous outcomes and risk difference (RD) with 95% CI for binary outcomes. For continuous outcomes measured on different scales, standardised mean difference (SMD) with 95% CI were used.

Heterogeneity was assessed using Cochran’s Q test (*χ*^2^) and the *I*^2^ statistic (32). A fixed-effect model was applied for I²<30%, a random-effects model was considered for I² between 30% (40%) and 70% (75%), and studies with I²≥70% were not combined. If meta-analysis was not feasible, a narrative synthesis was provided. For primary outcomes, subgroup analysis was conducted based on study characteristics, such as country, number of study centres, number of participants, insulin therapy, Intervention period and sensor usage pattern.

### 2.8 Publication bias assessment

Publication bias was assessed through visual inspection of funnel plot asymmetry (33) and Egger’s test, which evaluates the relationship between study size and effect magnitude (34). These analyses were performed as more than 10 trials were available, meeting the recommended threshold for such assessments.

## 3 Results

**Figure 1:**
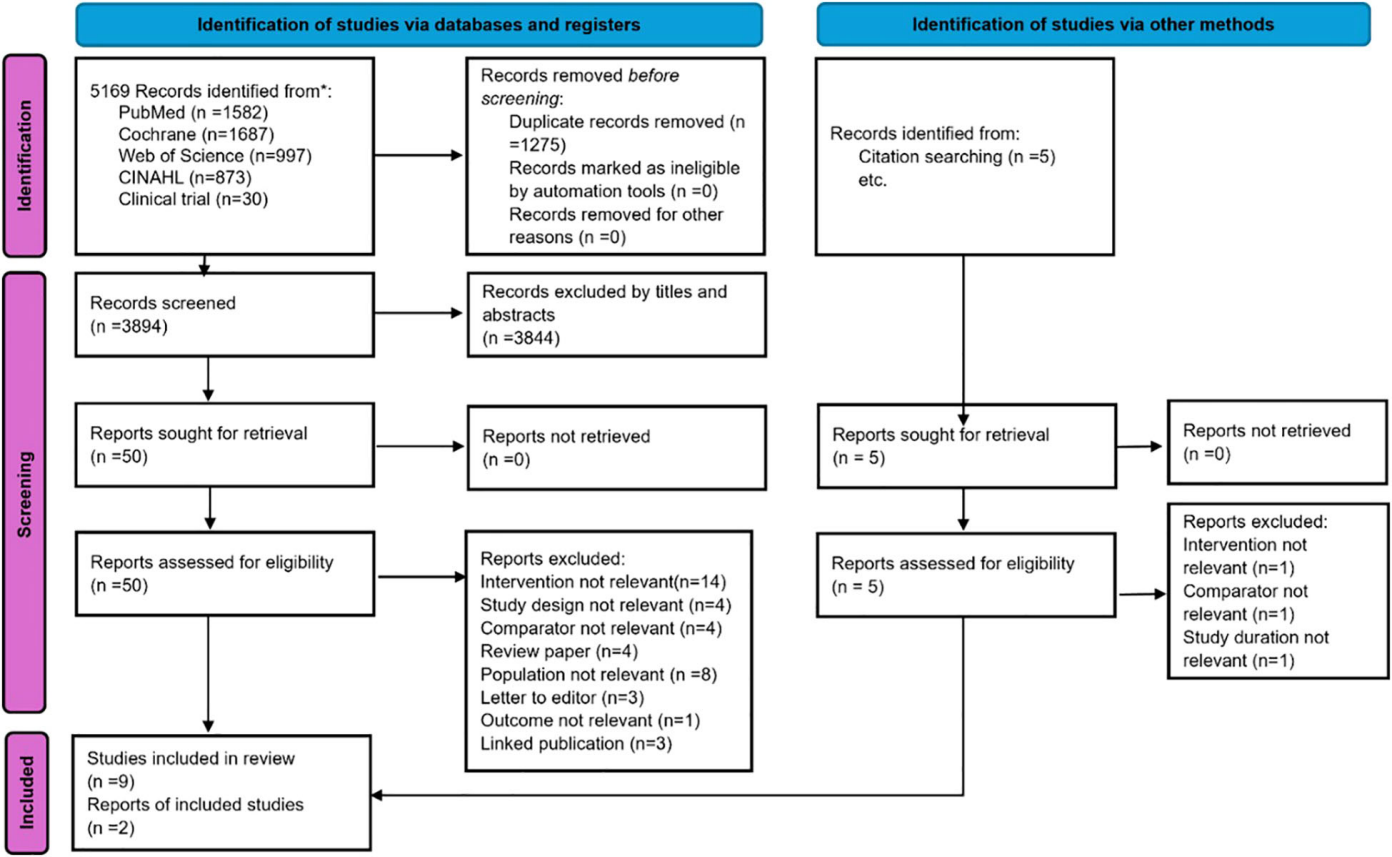
Selection of studies included in the meta-analysis.

The study selection process is illustrated in **Figure 1**, with an updated version provided in the **Supplementary Data 3**. The initial database search identified 5,169 potentially relevant citations from four databases and ClinicalTrials.gov, along with 5 additional citations retrieved through reference list screening. After removing 1,275 duplicates via electronic and manual screening, 3,894 records remained for title and abstract screening. Subsequently, the full texts of 55 studies were assessed for eligibility, and 44 studies were excluded for the following reasons: intervention not relevant (n=15), study design not relevant (n=4), comparator not relevant (n=5), review paper (n=4), population not relevant (n=8), letter to the editor (n=3), outcome not relevant (n=1), study duration not relevant (n=1), and linked publication (n=3). Ultimately, 11 randomised controlled trials (RCTs) met the inclusion criteria and were included in the final analysis (19, 36–45). The complete lists of included and excluded studies are provided in the **Supplementary Data 4** and **5**.

### 3.1 Study characteristics and risk of bias

The eleven RCTs were published between 2008 and 2023, comprising a total of 789 participants—437 in the rtCGM group and 352 in the SMBG group. The majority of studies were conducted in the United States (n=7) (19, 35, 37, 38, 40, 42, 43), while the rest were from Canada (n=2) (19, 41), France (n=1) (36), and Korea (n=2) (39, 44). Sample sizes ranged from 25 to 175 participants, with mean ages between 53 and 70 years (**Table 1**). Three studies included participants on oral hyperglycaemic agents (OHGAs) only (37, 39, 40), while eight studies included participants on both insulin and OHGAs (19, 35, 36, 38, 41–44). rtCGM was used in six studies continuously (19, 35, 37, 38, 41, 42) or intermittently (36, 39, 40, 43, 44) in five studies, using various systems, including Dexcom and Guardian devices, with study period of 12 to 56 weeks.

**Table 1:** Characteristics of included studies.

Study (Author, year)	Country	Number of centres	Number of participants (I^2^, C^3^)	Mean age (years)	DM^4^ treatment	Sensor usage pattern/ Intervention duration (Weeks)	Intervention (rtCGM) /Comparator (SMBG)	Outcome variables
Beck et al., 2017 (19)	US^1^ Canada	25	N=158 (I:79, C:79)	60.0	OHGAs^5^ + Insulin	Consistent (24)	Dexcom G4/ SMBG^6^	Change in HbA1c, high adherence to CGM^7^ used
Bergenstal et al., 2022 (35)	US^1^	1	N=114 (I:59, C:55)	59.1	OHGAs^5^ + Insulin	Consistent (16)	Dexcom G7/SMBG^6^	Change in HbA1c, hypoglycaemic, TIR^9^, glucose variability
Cosson et al., 2009	France	5	N=25 (I: 11, C:14)	57.2	OHGAs^5^ + Insulin	Intermittent (12)	GlucoDay/ SMBG^6^	Change in HbA1c, no major adverse events
Cox et al., 2020 (36)	US^1^	1	N=30 (I: 20, C:10)	53.3	OHGAs^5^	Consistent (20)	DexcomeG5/ SMBG^6^	Change in HbA1c improvement in QoL^8^ and reduced distress
Marten et al., 2021 (38) -Aleppo et al., 2021 (13)	US^1^	15	N=175 (I: 116, C:59)	57.0	OHGAs^5^ + Insulin	Consistent (32)	Dexcom G6/SMBG^6^	Change in HbA1c and glucose metrics
Moon et al., 2023 (39)	Korea	3	N=30 (I:15, C:15)	53.5	OHGAs^5^	Intermittent (24)	Guardian/ SMBG^6^	Change in HbA1c, no major adverse events
Price et al., 2018 (40)	US^1^	8	N=70 (I:46, C: 24)	70.0	OHGAs^5^	Intermittent (12)	SMBG^6^	Change in HbA1c, TIR^9^ without adverse events
Tang et al., 2014 (41) -Tildesley et al., 2013	Canada	1	N=40 (I: 20, C:20)	60.0	Insulin or OHGAs^5^ + insulin	Consistent (24)	Guardian/ SMBG^6^	Chang in HbA1c, BMI^10^, BP^11^ and treatment satisfaction
Taylor et al., 2019 (42)	US^1^	25	N=20 (I:10, C:10)	60.6	Insulin with or without OHGAs^5^	Consistently (24)	Dexcom G5/SMBG^6^	Change in HbA1c, glucose metrics, DTSQ^12^
Vigersky et al., 2012 (43) -Ehrhardt et al., 2011	US^1^	1	N=100 (I: 50, C: 50)	57.8	OHGAs^5^ + Insulin	Intermittent (52)	DexcomG7/ SMBG^6^	Change in HbA1c, Body weight, BP^11^ and diabetes-related distress
Yoo et al., 2008 (44)	Korea	4	N=65 (I: 32, C: 33)	56.0	OHGAs^5^ + Insulin	Intermittent (12)	Guardian/ SMBG^6^	Change in HbA1c, body weight, dietary intake and physical activity

^1^ United States

^2^ Intervention

^3^ Control

^4^ Diabetes Mellitus

^5^ Oral Hypoglycaemic Agents

^6^ Self-Monitoring Blood Glucose

^7^ Continuous Glucose Monitoring

^8^ Quality of Life

^9^ Time in Range

^10^ Body Mass Index

^11^ Blood Pressure

^12^ Diabetes Treatment Satisfaction Questionnaire

The risk of bias for the primary outcome of HbA1c was assessed using the Cochrane RoB 2.0 tool across 11 RCTs. Six studies (19, 35, 37–39, 42) were rated as having a low overall risk of bias, while five studies (36, 40, 41, 43, 44) were judged to have some concerns, primarily due to insufficient information on randomisation and allocation concealment, high attrition rates, or lack of trial registration. Overall, the evidence for HbA1c outcomes in rtCGM studies among individuals with type 2 diabetes was deemed to be of high methodological quality (see **Supplementary Data 6**).

### 3.2 Primary outcome

**Figure 2:**
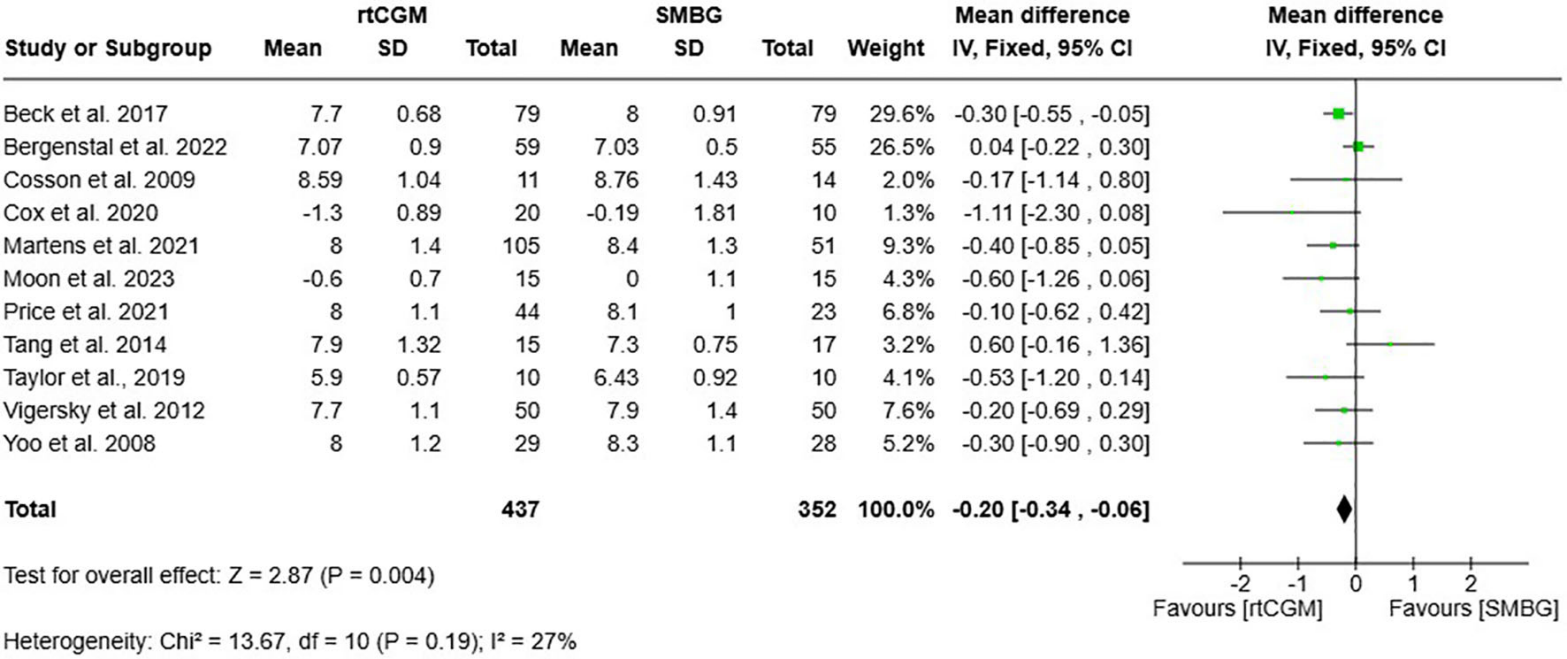
Forest plot and 95% CI of the pooled mean difference in HbA1c (%) between adults with type 2 diabetes using rtCGM and those using SMBG.

Meta-analysis of eleven RCTs (n=789) (19, 35–44) demonstrated a significant improvement in HbA1c levels with rtCGM compared to SMBG. The pooled mean difference was -0.20% (95% CI -0.34, -0.06; *p* = 0.004), favouring rtCGM, with low heterogeneity observed (I²=27%) (**Figure 2**).

#### 3.2.1 Subgroup analysis

A subgroup analysis was conducted to explore potential effect modifiers of HbA1c reduction following CGM intervention across various study characteristics (**Table 2**). Overall, the intervention group demonstrated a greater HbA1c reduction in studies conducted in Western countries (−0.17%, 95% CI −0.32, −0.03, *p* = 0.02) (19, 35–38, 40–42) compared to those in Asia (−0.44%, 95% CI −0.88, 0.01, *p* = 0.05) (39, 44), although the between-group difference was not statistically significant (*p* = 0.27). Studies (19, 36, 38–40, 42, 44) with multiple centres reported a significant HbA1c reduction (−0.33%, 95% CI −0.50, −0.15, *p* = 0.0003), while single-centre studies (35, 37, 41, 43) showed no effect, with a significant subgroup difference (*p* = 0.02). No significant subgroup differences were observed for number of participants (*p* = 0.71), insulin use (*p* = 0.34), intervention period (*p* = 0.83), or sensor usage pattern (*p* = 0.79).

**Table 2:** Subgroup analysis based on study characteristics by HbA1c.

Characteristics	Subgroup	Reference ID	N	Mean difference	95% CI		Z(p)	Test for subgroup sifference (P)
					Lower limit	Upper limit		
Country	Asia	(39, 44)	87	-0.44	-0.88	0.01	1.93 (0.05)	0.27
	Western	(19, 35–38, 40–43)	702	-0.17	-0.32	-0.03	2.39(0.02)	
Number of Study Centres	Single	(35, 37, 41, 43)	276	0.00	-0.22	0.22	0.00 (1.00)	0.02
	Multiple	(19, 36, 38–40, 42, 44)	513	-0.33	-0.50	-0.15	3.66(0.0003)	
Number of participants	<100	(36, 37, 39–42, 44)	261	-0.26	-0.58	-0.05	1.63 (0.10)	0.71
	≥100	(19, 35, 38, 43)	528	-0.19	-0.40	0.02	1.74 (0.08)	
Insulin Therapy	No	(37, 39, 40)	127	-0.44	-0.94	0.07	1.70(0.09)	0.34
	Yes	(19, 35–38, 41–44)	662	-0.18	-0.36	0.01	2.13 (0.03)	
Intervention period	12 weeks	(36, 40, 43)	149	-0.18	-0.55	0.18	0.99 (0.32)	0.83
	>12 weeks	(19, 35, 37–39, 41, 42, 44)	640	-0.21	-0.38	0.05	2.10(0.04)	
Sensor Usage Pattern	Intermittently	(36, 39, 40, 43, 44)	279	-0.26	-0.53	0.01	1.88 (0.06)	0.79
	Consistently	(19, 35, 37, 38, 41, 42)	510	-0.20	-0.48	0.08	1.42(0.15)	

### 3.3 Secondary outcomes

#### 3.3.1 Glucose metrics

**Figure 3:**
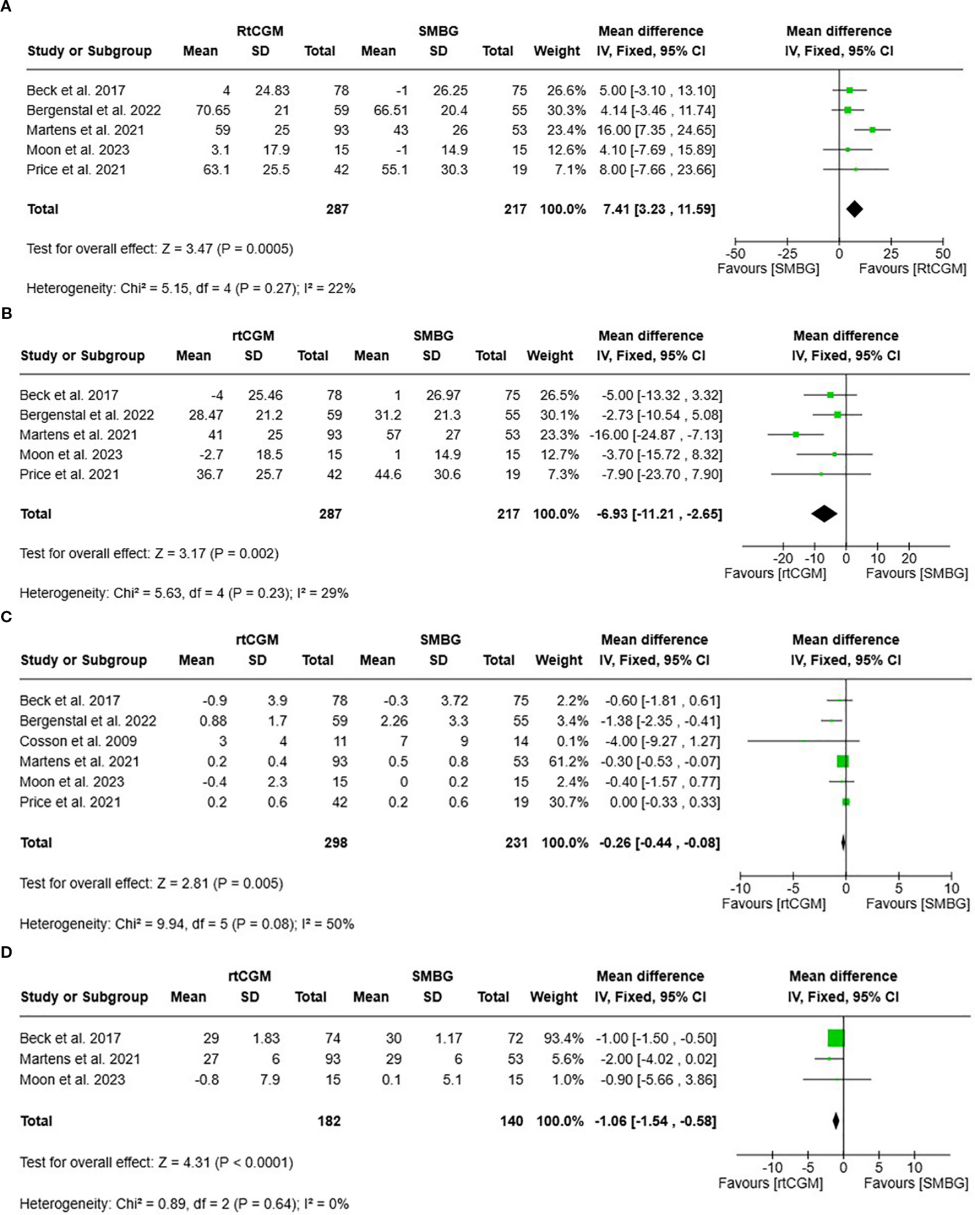
**(A)** Forest plot and 95% CI of TIR, which is categorised as glucose readings between 3.9 to 10mmol/L in individuals with type 2 diabetes using rtCGM compared with SMBG. **(B)** Forest plot and 95% CI of TAR, which is categorised as glucose readings above 10mmol/L in individuals with type 2 diabetes using rtCGM compared with SMBG. **(C)** Forest plot and 95% CI of TBR, which is categorised as glucose readings below 3.9mmol/L in individuals with type 2 diabetes using rtCGM compared with SMBG. **(D)** Forest plot and 95% CI of glucose variability (Coefficient of Variation) in individuals with type 2 diabetes using rtCGM compared with SMBG.

A meta-analysis of studies rtCGM with SMBG demonstrated significant improvements across multiple glycaemic metrics in favour of rtCGM. Based on five studies (n = 504) (19, 35, 38–40), rtCGM significantly increased TIR compared to SMBG, with a pooled MD of 7.41% (95% CI 3.23, 11.59, *P* = 0.0005; I² = 22%) (**Figure 3A**). Five studies (n = 504) (19, 35, 38–40) showed that rtCGM significantly reduced TAR, with a pooled MD -6.93% (95% CI -11.21, -2.65, *P* = 0.002; I² = 29%) (**Figure 3B**). Six studies (n = 529) (19, 35, 36, 38–40) found a significant reduction in TBR with rtCGM, with a pooled MD of **-**0.26% (95% CI -0.44, -0.08, *P* = 0.005; I² = 50%) (**Figure 3C**). Three studies (n = 322) (19, 38, 39) reported lower glucose variability with rtCGM use, with a pooled MD of -1.06% (95% CI -1.54, -0.58, *P* < 0.0001; I² = 0%) (**Figure 3D**).

#### 3.3.2 Cardiometabolic parameters

The impact of rtCGM on anthropometric outcomes was evaluated across multiple studies. Pooled analysis of seven studies (n = 620) (19, 35, 38, 39, 42–44) comparing body weight between rtCGM and SMBG users showed no statistically significant difference, with a MD of –0.95 kg (95% CI –2.37, 0.48; *P* = 0.19; I² = 32%). Similarly, five studies (n = 430) (35, 38, 41, 43, 44) assessed changes in BMI and found no significant difference between groups, with a MD of –0.68 (95% CI –1.73, 0.38; *P* = 0.21; I² = 41%) (**Supplementary Data 9**).

Pooled analysis showed no significant differences between rtCGM and SMBG in terms of lipid profiles. For LDL, HDL and triglycerides, the MD was –0.02 mmol/L (95% CI -0.19, 0.15; *P* = 0.85; I² = 20%) (37–39, 42, 44), 0.02 mmol/L (95% CI –0.06, 0.10; *P* = 0.69; I² = 69%), and 0.01 mmol/L; 95% CI –0.32, 0.35; *P* = 0.94; I² = 47%) (37, 39, 42, 44) respectively (**Supplementary Data 10**).

The effects of rtCGM on blood pressure were assessed across six studies (n = 391) (35, 38, 39, 41, 43). For SBP, pooled analysis of six studies (rtCGM: 214, SMBG: 177) showed no significant difference between groups, with a MD of 0.19 mmHg (95% CI –2.62, 2.99; *P* = 0.90; I² = 36%). Similarly, for DBP, the pooled MD was 0.37 mmHg (95% CI –1.38, 2.13; *P* = 0.68; I² = 0%), also indicating no statistically significant effect (**Supplementary Data 11**).

#### 3.3.3 Self-care behaviours

Analysis of self-care behaviours compared rtCGM and SMBG across multiple domains. For dietary behaviours, pooled data from two studies (n=87) (37, 44) showed a non-significant trend in total calorie reduction favouring rtCGM (MD = -71.68 kcal; 95% CI -199.25, 55.89; p=0.27; I²=0%). Carbohydrate intake, assessed in one study (n=30) (37), demonstrated significantly lower consumption in the rtCGM group (MD = -79.10g; 95% CI -156.3, -1.86; p=0.04). Physical activity, reported in a single study (n=57) (44), showed significantly higher exercise duration among rtCGM users (MD = 111.60 minutes; 95% CI 10.94, 212.26; p=0.03). Regarding monitoring frequency, rtCGM users performed significantly fewer daily glucose measurements compared to SMBG users (MD = -0.99 times/day; 95% CI -1.22, -0.77; p<0.00001; I²=0%) (38, 39). rtCGM users demonstrated improvement in diabetes knowledge (37) (SMD = 1.65; 95% CI 0.77, 2.53; p=0.0002) and self-management readiness (SMD = 0.69; 95% CI 0.15, 1.23; p=0.01; I²=0%) (37, 39) (**Supplementary Data 12**).

#### 3.3.4 Diabetes treatment satisfaction

Treatment satisfaction was reported in two studies (n = 62; rtCGM: 35, SMBG: 27). The pooled analysis showed no statistically significant difference in satisfaction scores between rtCGM and SMBG groups, with a SMD of –0.48 (95% CI –2.69,1.73; *P* = 0.67, I²=94%) (37, 41). The CGM Satisfaction Scale demonstrated high levels of user satisfaction among individuals using real-time CGM, with mean scores reaching 4.4 out of 5 (19, 38).

#### 3.3.5 Health-related quality of life

In terms of HRQoL, data from Beck et al. (2017) (n = 150) also showed no significant difference between groups, with a SMD of 0.00 (95% CI –0.32, 0.32; *P* = 1.00) (19). No heterogeneity was observed (**Supplementary Data 12**).

#### 3.3.6 Adverse events

Four studies (n = 268) reported skin reactions. The pooled analysis showed no statistically significant difference between rtCGM and SMBG groups (RD: 0.01, 95% CI –0.02, 0.05; *P* = 0.41; I² = 54%) (36, 38–40). Two studies (n = 176) assessed general hypoglycaemia events. The pooled RD was 0.02 (95% CI –0.02,0.07; *P* = 0.31; I² = 96%), showing no significant difference (37, 38). One study reported on severe hypoglycaemia and diabetic ketoacidosis (DKA) (38). Both outcomes showed no significant difference between groups (RD for each: –0.01, 95% CI –0.05, 0.03; *P* = 0.71) (**Supplementary Data 13**).

#### 3.3.7 Publication bias

Assessment of publication bias through funnel plot (**Supplementary Data 15**) visualisation and Egger’s regression test (z = -0.4404, p = 0.6597) revealed no significant asymmetry in HbA1c outcomes, indicating minimal publication bias. Analyses were performed using RStudio (version 4.4.3).

#### 3.3.8 Quality of evidence assessment

GRADE certainty of the evidence ranged from low to moderate (**Supplementary Data 16**).

## 4 Discussion

This systematic review and meta-analysis demonstrated that rtCGM confers advantages over SMBG in adults with type 2 diabetes, improving glycaemic control and multiple CGM-derived glucose metrics while maintaining a comparable safety profile. Beyond traditional glycaemic outcomes, rtCGM appears to support daily glycaemic stability and aspects of diabetes self-management, underscoring its relevance in contemporary diabetes care.

The primary outcome, HbA1c, showed a statistically significant reduction of 0.20% with rtCGM compared with SMBG, with low heterogeneity (I² = 27%), indicating consistency across included studies. This finding aligns with prior randomised trials demonstrating improved glycaemic control with rtCGM, including among insulin-treated individuals with type 2 diabetes (20–24, 45). However, the magnitude of HbA1c reduction did not meet the 0.5% threshold considered clinically meaningful by current guidelines from the American Diabetes Association and the National Institute for Health and Care Excellence (46, 47).

Despite this, modest reductions in HbA1c may still be clinically relevant in specific patient subgroups. For individuals near glycaemic targets, minor incremental improvements may help maintain control and delay treatment intensification. Similarly, in patients at higher risk of hypoglycaemia or those with early dysglycaemia, modest changes in HbA1c may reflect meaningful reductions in glycaemic excursions not fully captured by HbA1c alone. Three small-sample studies (37, 39, 42) reported HbA1c reductions exceeding 0.5%. Although these findings may have limited generalisability, they potentially demonstrate the right ingredients necessary for more effective HbA1c reduction - greater population homogeneity and closer monitoring with tailored interventions amongst these studies.

Subgroup analysis by region showed a numerically larger HbA1c reduction in Asian studies (−0.44%) compared with Western studies (−0.17%), but the subgroup difference was not statistically significant (P = 0.27) and should be interpreted cautiously given the substantial imbalance in sample size (87 vs. 702 participants). Nevertheless, this pattern raises the possibility that ethnic, cultural, or healthcare-system factors may modulate the effectiveness of rtCGM. Differences in dietary patterns, such as higher carbohydrate intake and greater glycaemic variability, commonly observed in Asian populations, may increase the utility of real-time glucose feedback. Variations in healthcare delivery models, diabetes education, affordability, and patient engagement with technology, as well as broader socioeconomic factors such as access to devices and digital health literacy, may further influence adherence and behavioural responses to rtCGM.

Importantly, rtCGM demonstrated clinically meaningful improvements in CGM-derived metrics beyond HbA1c. TIR increased by 7.41%, and TAR decreased by 6.93%, both exceeding the 5% threshold considered clinically relevant according to international consensus recommendations (48). These findings indicate improved daily glycaemic stability and reduced exposure to hyperglycaemia. Although the reduction in TBR was modest and did not meet clinically meaningful thresholds, it suggests that rtCGM improves overall glycaemic profiles without increasing the risk of hypoglycaemia. Improvements in glucose variability, an essential but often underreported parameter, further support the value of rtCGM in stabilising day-to-day glucose fluctuations. Collectively, these findings reinforce emerging evidence that composite CGM metrics may be more sensitive and clinically actionable indicators of intervention benefit than HbA1c alone, particularly in early dysglycaemia or prediabetes, where HbA1c may underestimate glycaemic abnormalities (49, 50).

In contrast, rtCGM did not demonstrate a significant effect on cardiometabolic outcomes, including body weight, body mass index, lipid profiles, or blood pressure. These findings are consistent with previous reviews (51, 52) and suggest that cardiometabolic parameters are influenced by multiple factors beyond glucose monitoring alone, including diet, physical activity, medication regimens, and duration of follow-up. Longer-term studies or multifactorial interventions may be required to determine whether improved glycaemic awareness through rtCGM translates into cardiometabolic benefits.

Beyond clinical outcomes, rtCGM appeared to positively influence aspects of self-care behaviour and readiness for diabetes self-management. Continuous visibility of glucose trends and immediate feedback may enhance patient empowerment, self-efficacy, and informed decision-making. Some studies (37, 43) reported favourable lifestyle changes, including reduced carbohydrate intake and increased physical activity, although these findings were limited to individual trials. rtCGM users also performed fewer daily fingerstick checks, reflecting greater convenience without compromising glycaemic control. However, pooled analyses did not demonstrate consistent improvements in treatment satisfaction or quality of life, despite high satisfaction scores reported in some individual studies (38). Heterogeneity in measurement instruments, follow-up duration, and patient expectations may partly explain these mixed findings.

The safety profile of rtCGM was comparable to SMBG across the included studies. Although minor adverse events such as skin reactions and hypoglycaemia were reported more frequently among rtCGM users, these differences were not statistically significant, and data on severe hypoglycaemia and diabetic ketoacidosis were limited. Given that a substantial proportion of participants were insulin-treated, the safety of hypoglycaemia is clinically essential. The modest reduction in TBR, together with real-time alerts and glucose trend information, suggests that rtCGM may facilitate earlier detection and mitigation of impending hypoglycaemia without increasing risk.

Finally, it is also important to frame rtCGM within the rapidly evolving landscape of digital diabetes management. Emerging evidence indicates that integrating CGM with artificial intelligence–driven analytics and personalised feedback systems may further enhance clinical utility by enabling pattern recognition, predicting dysglycaemic events, and tailoring behavioural or therapeutic interventions. Evidence from prediabetes populations suggests that such integrative CGM-artificial intelligence (AI) approaches may refine intervention timing and personalisation (53), with potential relevance for individuals with early type 2 diabetes or modest HbA1c elevations.

## 5 Strengths and limitations

This review has several strengths. It is among the first to comprehensively evaluate both clinical and behavioural outcomes of rtCGM compared with SMBG in adults with type 2 diabetes using meta-analytic methods. The inclusion of multiple CGM-derived glucose metrics alongside self-care behaviours, treatment satisfaction, and quality of life provides a holistic assessment of the impact of rtCGM. In addition, most included studies demonstrated a low risk of bias, strengthening confidence in the robustness of the findings.

Several limitations should be acknowledged. Heterogeneity was observed in psychosocial outcomes, likely reflecting differences in intervention duration, educational components, user interface design, and outcome measurement instruments. The predominance of studies conducted in Western countries and the exclusion of non-English publications may limit global generalisability and introduce language bias. Furthermore, data on severe hypoglycaemia, diabetic ketoacidosis, long-term diabetes-related complications, and cost-effectiveness were limited. These represent critical evidence gaps, particularly for informing health policy, reimbursement decisions, and large-scale implementation of rtCGM in routine care.

## 6 Conclusion

In conclusion, rtCGM provides significant advantages over SMBG in improving glycaemic control and CGM-derived metrics in adults with type 2 diabetes, while maintaining a comparable safety profile. Although the reduction in HbA1c was modest, clinically meaningful improvements in time in range, time above range, and glucose variability highlight the added value of rtCGM beyond HbA1c alone. The observed behavioural benefits further suggest that rtCGM can support more proactive and personalised diabetes self-management, particularly among insulin-treated individuals.

Future research should prioritise longer-term studies evaluating diabetes-related complications and cost-effectiveness, include more geographically and ethnically diverse populations, and identify patient subgroups most likely to benefit from rtCGM. Standardisation of psychosocial outcome measures and exploration of integrative CGM–AI approaches will be essential to fully define the clinical, economic, and policy-relevant role of rtCGM in modern diabetes care.

## Data Availability

The original contributions presented in the study are included in the article/**Supplementary Material**. Further inquiries can be directed to the corresponding author.
