# Supplementary material for: Impact of real-time continuous glucose monitoring on glycaemic control in adults with type 2 diabetes: systematic review and meta-analysis
Source: Front Endocrinol (Lausanne). 2026 Jan 23;16:1761579. doi: 10.3389/fendo.2025.1761579 (PMC12875926; doi:10.3389/fendo.2025.1761579)
Supplement: Supplementary file 1 [file DataSheet1.docx]

**Supplementary Data**

| Supplementary Data Number | Content |
| --- | --- |
| 1 | PRISMA 2020 Checklist |
| 2 | The Details of Search Terms |
| 3 | PRISMA 2020 Flow Diagram Updated from Oct 2024 to Jul 2025 |
| 4 | The list of Included Studies |
| 5 | The list of Excluded Studies |
| 6 | Risk of Bias Summary |
| 7 | Forest Plot of the Effect of rtCGM on HbA1c Compared with SMBG |
| 8 | Forest Plot of the Effect of rtCGM on Glucose Metrics (TIR, TAR, TBR, glucose variability) Compared with SMBG |
| 9 | Forest Plot of the Effect of rtCGM on Cardiometabolic Parameters (Body Weight and BMI) Compared with SMBG |
| 10 | Forest Plot of the Effect of rtCGM on Cardiometabolic Parameters (LDL, HDL and TG) Compared with SMBG |
| 11 | Forest Plot of the Effect of rtCGM on Cardiometabolic Parameters (SBP and DBP) Compared with SMBG |
| 12 | Forest Plot of the Effect of rtCGM on Self-Care Behaviour Compared with SMBG |
| 13 | Forest Plot of the Effect of rtCGM on Psychological Outcomes Compared with SMBG |
| 14 | Risk of Adverse Events of rtCGM Compared with SMBG |
| 15 | Funnel Plot for Change in HbA1C Levels Comparing rtCGM with SMBG |
| 16 | GRADE Summary of Findings |

**Supplementary Data 1: PRISMA 2020 Checklist**

| **Section and Topic** | **Item #** | **Checklist item** | **Location where item is reported** |
| --- | --- | --- | --- |
| **TITLE** | | |  |
| Title | 1 | Identify the report as a systematic review. | Title |
| **ABSTRACT** | | |  |
| Abstract | 2 | See the PRISMA 2020 for Abstracts checklist. | Study Abstract |
| **INTRODUCTION** | | |  |
| Rationale | 3 | Describe the rationale for the review in the context of existing knowledge. | Introduction |
| Objectives | 4 | Provide an explicit statement of the objective(s) or question(s) the review addresses. | Aims |
| **METHODS** | | |  |
| Eligibility criteria | 5 | Specify the inclusion and exclusion criteria for the review and how studies were grouped for the syntheses. | Methods |
| Information sources | 6 | Specify all databases, registers, websites, organisations, reference lists and other sources searched or consulted to identify studies. Specify the date when each source was last searched or consulted. | Methods |
| Search strategy | 7 | Present the full search strategies for all databases, registers and websites, including any filters and limits used. | Supplementary Data 2 |
| Selection process | 8 | Specify the methods used to decide whether a study met the inclusion criteria of the review, including how many reviewers screened each record and each report retrieved, whether they worked independently, and if applicable, details of automation tools used in the process. | Methods |
| Data collection process | 9 | Specify the methods used to collect data from reports, including how many reviewers collected data from each report, whether they worked independently, any processes for obtaining or confirming data from study investigators, and if applicable, details of automation tools used in the process. | Methods |
| Data items | 10a | List and define all outcomes for which data were sought. Specify whether all results that were compatible with each outcome domain in each study were sought (e.g. for all measures, time points, analyses), and if not, the methods used to decide which results to collect. | Methods |
|  | 10b | List and define all other variables for which data were sought (e.g. participant and intervention characteristics, funding sources). Describe any assumptions made about any missing or unclear information. | Methods |
| Study risk of bias assessment | 11 | Specify the methods used to assess risk of bias in the included studies, including details of the tool(s) used, how many reviewers assessed each study and whether they worked independently, and if applicable, details of automation tools used in the process. | Methods |
| Effect measures | 12 | Specify for each outcome the effect measure(s) (e.g. risk ratio, mean difference) used in the synthesis or presentation of results. | Methods |
| Synthesis methods | 13a | Describe the processes used to decide which studies were eligible for each synthesis (e.g. tabulating the study intervention characteristics and comparing against the planned groups for each synthesis (item #5)). | Results |
|  | 13b | Describe any methods required to prepare the data for presentation or synthesis, such as handling of missing summary statistics, or data conversions. | Methods |
|  | 13c | Describe any methods used to tabulate or visually display results of individual studies and syntheses. | NA |
|  | 13d | Describe any methods used to synthesize results and provide a rationale for the choice(s). If meta-analysis was performed, describe the model(s), method(s) to identify the presence and extent of statistical heterogeneity, and software package(s) used. | Methods |
|  | 13e | Describe any methods used to explore possible causes of heterogeneity among study results (e.g. subgroup analysis, meta-regression). | Methods |
|  | 13f | Describe any sensitivity analyses conducted to assess robustness of the synthesized results. | NA |
| Reporting bias assessment | 14 | Describe any methods used to assess risk of bias due to missing results in a synthesis (arising from reporting biases). | Methods |
| Certainty assessment | 15 | Describe any methods used to assess certainty (or confidence) in the body of evidence for an outcome. | Methods |
| **RESULTS** | | |  |
| Study selection | 16a | Describe the results of the search and selection process, from the number of records identified in the search to the number of studies included in the review, ideally using a flow diagram. | Figure 1 |
|  | 16b | Cite studies that might appear to meet the inclusion criteria, but which were excluded, and explain why they were excluded. | Supplementary Date 5 |
| Study characteristics | 17 | Cite each included study and present its characteristics. | Table 1, Supplementary Data 4 |
| Risk of bias in studies | 18 | Present assessments of risk of bias for each included study. | Supplementary Data 6 |
| Results of individual studies | 19 | For all outcomes, present, for each study: (a) summary statistics for each group (where appropriate) and (b) an effect estimate and its precision (e.g. confidence/credible interval), ideally using structured tables or plots. | Figures 2, 3 |
| Results of syntheses | 20a | For each synthesis, briefly summarise the characteristics and risk of bias among contributing studies. | Results |
|  | 20b | Present results of all statistical syntheses conducted. If meta-analysis was done, present for each the summary estimate and its precision (e.g. confidence/credible interval) and measures of statistical heterogeneity. If comparing groups, describe the direction of the effect. | Figures 2, 3 |
|  | 20c | Present results of all investigations of possible causes of heterogeneity among study results. | Results |
|  | 20d | Present results of all sensitivity analyses conducted to assess the robustness of the synthesized results. | NA |
| Reporting biases | 21 | Present assessments of risk of bias due to missing results (arising from reporting biases) for each synthesis assessed. | Results |
| Certainty of evidence | 22 | Present assessments of certainty (or confidence) in the body of evidence for each outcome assessed. | Results, Supplementary Data 16 |
| **DISCUSSION** | | |  |
| Discussion | 23a | Provide a general interpretation of the results in the context of other evidence. | Discussion |
|  | 23b | Discuss any limitations of the evidence included in the review. | Discussion |
|  | 23c | Discuss any limitations of the review processes used. | Discussion |
|  | 23d | Discuss implications of the results for practice, policy, and future research. | Discussion |
| **OTHER INFORMATION** | | |  |
| Registration and protocol | 24a | Provide registration information for the review, including register name and registration number, or state that the review was not registered. | Abstract |
|  | 24b | Indicate where the review protocol can be accessed, or state that a protocol was not prepared. | Abstract |
|  | 24c | Describe and explain any amendments to information provided at registration or in the protocol. | NA |
| Support | 25 | Describe sources of financial or non-financial support for the review, and the role of the funders or sponsors in the review. | Funding |
| Competing interests | 26 | Declare any competing interests of review authors. | Conflicts of Interest |
| Availability of data, code and other materials | 27 | Report which of the following are publicly available and where they can be found: template data collection forms; data extracted from included studies; data used for all analyses; analytic code; any other materials used in the review. | Data Availability |

**Appendix 2: The Details of Search Terms**

| **PubMed** |  |
| --- | --- |
| #1 | "Diabetes Mellitus, Type 2"[Mesh] |
| #2 | (Diabet*[Title/Abstract] AND ("type 2"[Title/Abstract] OR type2[Title/Abstract] OR "type ii"[Title/Abstract] OR typeii [Title/Abstract] OR T2[Title/Abstract] OR Noninsulin Dependent [Title/Abstract] OR Non-insulin Dependent [Title/Abstract])) OR T2DM[Title/Abstract] OR NIDDM[Title/Abstract] |
| #3 | #1 OR #2 |
| #4 | ("Blood Glucose"[Mesh] AND continuous[ti]) OR ("Continuous Glucose Monitoring"[Mesh]) |
| #5 | Continuous Glucose Monitor*[Title/Abstract] OR " Ambulatory Glucose Monitor*" [Title/Abstract] OR " Flash Glucose Monitor*" [Title/Abstract] OR "CGMS" [Title/Abstract] OR "Intermittently Scanned CGM" [Title/Abstract] OR "Real-time CGM" [Title/Abstract] OR rtCGM [Title/Abstract] OR isCGM [Title/Abstract] |
| #6 | #3 AND (#4 OR #5) |
| #7 | (randomized controlled trial[pt] OR controlled clinical trial[pt] OR randomized[tiab] OR placebo[tiab] OR drug therapy[sh] OR randomly[tiab] OR trial[tiab] OR groups[tiab] NOT (animals [mh] NOT humans [mh])) |
| #8 | #6 AND #7 |

| **COCHRANE** |  |
| --- | --- |
| #1 | MeSH descriptor: [Diabetes Mellitus, Type 2] explode all trees |
| #2 | ((Diabet* AND ("type 2" OR type2 OR "type ii" OR typeii OR T2 OR Noninsulin Dependent OR Non-insulin Dependent)) OR T2DM OR NIDDM):ti,ab |
| #3 | #1 OR # 2 |
| #4 | MeSH descriptor: [Blood Glucose] explode all trees |
| #5 | Continuous:ti |
| #6 | #4 AND #5 |
| #7 | MeSH descriptor: [Continuous Glucose Monitoring] explode all trees |
| #8 | ("continuous glucose monitoring" OR ("continuous" AND "glucose" AND "monitoring" OR "continuous glucose monitoring") OR " CGM"): ti,ab |
| #9 | #6 OR #7 OR #8 |
| #10 | #3 AND #9 |

| **Web of Science** |  | |
| --- | --- | --- |
| 1 | TS= (((("Diabetes Mellitus*" OR Diabetes*) NEAR/3 ("type 2" OR type2 OR "type ii" OR typeii OR “Noninsulin Dependent” OR “Non-insulin Dependent”)) OR T2DM OR NIDDM) NEAR/15 ("Continuous Glucose Monitor*" OR "Ambulatory Glucose Monitor*” OR "Flash Glucose Monitor*" OR CGMS OR "Intermittently Scanned CGM" OR “Real-time CGM” OR rtCGM OR isCGM) ) | |
| **CINAHL** |  |  |
| S1 | (MH "Diabetes Mellitus, Type 2") |  |
| S2 | (MH "Diabetes Mellitus, Type 2") OR TI ( ((Diabet* AND ("type 2" OR type2 OR "type ii" OR typeii OR T2 OR Noninsulin Dependent OR Non-insulin Dependent)) OR T2DM OR NIDDM) ) OR AB ( ((Diabet* AND ("type 2" OR type2 OR "type ii" OR typeii OR T2 OR Noninsulin Dependent OR Non-insulin Dependent)) OR T2DM OR NIDDM) ) |  |
| S3 | (MH "Blood Glucose") |  |
| S4 | (MH "Blood Glucose") AND TI continuous |  |
| S5 | (MH "Continuous Glucose Monitoring") |  |
| S6 | (MH "Continuous Glucose Monitoring") OR TI ( "Continuous Glucose Monitor*" OR "Ambulatory Glucose Monitor*" OR "Flash Glucose Monitor*" OR CGMS OR "Intermittently Scanned CGM" OR “Real-time CGM” OR rtCGM OR isCGM ) OR AB ( "Continuous Glucose Monitor*" OR "Ambulatory Glucose Monitor*" OR "Flash Glucose Monitor*" OR CGMS OR "Intermittently Scanned CGM" OR “Real-time CGM” OR rtCGM OR isCGM ) |  |
| S7 | S4 OR S5 OR S6 |  |
| S8 | S2 AND S7 |  |

**Appendix 3: PRISMA 2020 Flow Diagram Updated from OCT 2024 to Jul 2025**

**
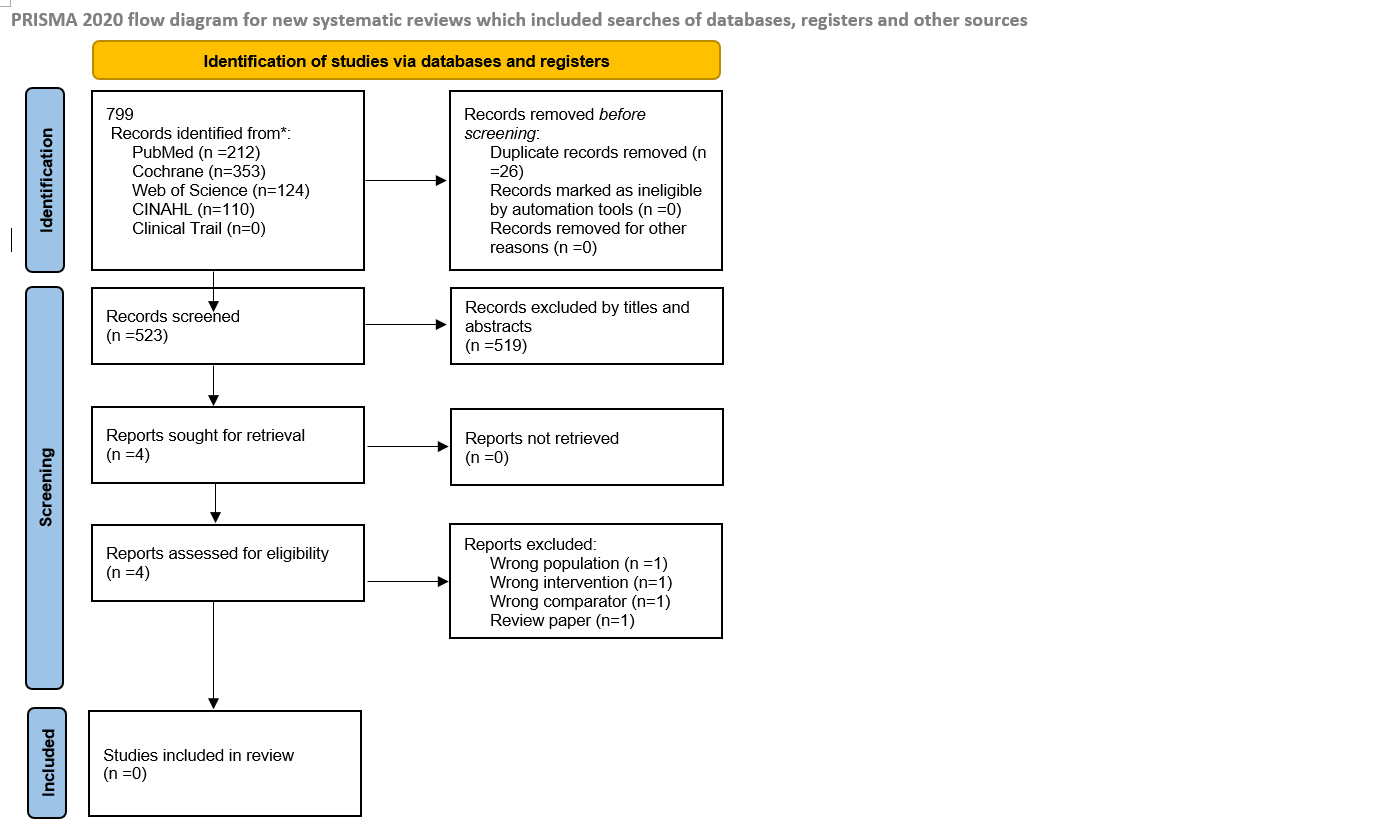
**

**Appendix 4. The list of Included studies**

| **Year** | **Identification** | **Reference for Included Studies** |
| --- | --- | --- |
| 2017 | Databases and registers | Beck, R. W., Riddlesworth, T. D., Ruedy, K., Ahmann, A., Haller, S., Kruger, D., McGill, J. B., Polonsky, W., Price, D., Aronoff, S., Aronson, R., Toschi, E., Kollman, C., & Bergenstal, R. (2017). Continuous glucose monitoring versus usual care in patients with type 2 diabetes receiving multiple daily insulin injections: A randomized trial. Annals of Internal Medicine, 167(6), 365-374. <https://doi.org/10.7326/M16-2855> |
| 2022 | Databases and registers | Bergenstal, R. M., Mullen, D. M., Strock, E., Johnson, M. L., & Xi, M. X. (2022). Randomized comparison of self-monitored blood glucose (BGM) versus continuous glucose monitoring (CGM) data to optimize glucose control in type 2 diabetes. Journal of Diabetes Complications, 36(3), Article 108106. <https://doi.org/10.1016/j.jdiacomp.2021.108106> |
| 2009 | Databases and registers | Cosson, E., Hamo-Tchatchouang, E., Dufaitre-Patouraux, L., Attali, J. R., Pariès, J., & Schaepelynck-Bélicar, P. (2009). Multicentre, randomised, controlled study of the impact of continuous sub-cutaneous glucose monitoring (GlucoDay) on glycaemic control in type 1 and type 2 diabetes patients. Diabetes & Metabolism, 35(4), 312-318. <https://doi.org/10.1016/j.diabet.2009.02.006> |
| 2020 | Databases and registers | Cox, D. J., Banton, T., Moncrief, M., Conaway, M., Diamond, A., & McCall, A. L. (2020). Minimizing glucose excursions (GEM) with continuous glucose monitoring in type 2 diabetes: A randomized clinical trial. Journal of the Endocrine Society, 4(11), Article bvaa118. <https://doi.org/10.1210/jendso/bvaa118> |
| 2021  2023 | Databases and registers  Databases and registers | Martens, T., Beck, R. W., Bailey, R., Ruedy, K. J., Calhoun, P., Peters, A. L., Pop-Busui, R., Philis-Tsimikas, A., Bao, S., Umpierrez, G., Davis, G., Kruger, D., Bhargava, A., Young, L., McGill, J. B., Aleppo, G., Nguyen, Q. T., Orozco, I., Biggs, W., ...Bergenstal, R. M. (2021). Effect of continuous glucose monitoring on glycemic control in patients with type 2 diabetes treated with basal insulin: A randomized clinical trial. JAMA, 325(22), 2262-2272. <https://doi.org/10.1001/jama.2021.7444>  Moon, S. J., Kim, K. S., Lee, W. J., Lee, M. Y., Vigersky, R., & Park, C. Y. (2023). Efficacy of intermittent short-term use of a real-time continuous glucose monitoring system in non-insulin-treated patients with type 2 diabetes: A randomized controlled trial. Diabetes, Obesity and Metabolism, 25(1), 110-120. <https://doi.org/10.1111/dom.14852> |
| **Year** | **Identification** | **Reference for Included Studies** |
| 2021 | Databases and registers | Price, D. A., Deng, Q., Kipnes, M., & Beck, S. E. (2021). Episodic real-time CGM use in adults with type 2 diabetes: Results of a pilot randomized controlled trial. *Diabetes Therapy, 12*(7), 2089–2099. <https://doi.org/10.1007/s13300-021-01086-y> |
| 2019 | Other Methods | Taylor, P. J., Thompson, C. H., Luscombe-Marsh, N. D., Wycherley, T. P., Wittert, G., & Brinkworth, G. D. (2019). Efficacy of real-time continuous glucose monitoring to improve effects of a prescriptive lifestyle intervention in type 2 diabetes: A pilot study. *Diabetes Therapy, 10*(2), 509–522. <https://doi.org/10.1007/s13300-019-0572-z> |
| 2014 | Databases and registers | Tang, T. S., Digby, E. M., Wright, A. M., Chan, J. H., Mazanderani, A. B., Ross, S. A., Tildesley, H. G., Lee, A. M., White, A. S., & Tildesley, H. D. (2014). Real-time continuous glucose monitoring versus internet-based blood glucose monitoring in adults with type 2 diabetes: A study of treatment satisfaction. Diabetes Research and Clinical Practice, 106(3), 481-486. <https://doi.org/10.1016/j.diabres.2014.09.050> |
| 2012 | Databases and registers | Vigersky, R. A., Fonda, S. J., Chellappa, M., Walker, M. S., & Ehrhardt, N. M. (2012). Short- and long-term effects of real-time continuous glucose monitoring in patients with type 2 diabetes. *Diabetes Care, 35*(1), 32–38. <https://doi.org/10.2337/dc11-1438> |
| 2008 | Databases and registers | Yoo, H. J., An, H. G., Park, S. Y., Ryu, O. H., Kim, H. Y., Seo, J. A., Hong, E. G., Shin, D. H., Kim, Y. H., Kim, S. G., Choi, K. M., Park, I. B., Yu, J. M., & Baik, S. H. (2008). Use of a real-time continuous glucose monitoring system as a motivational device for poorly controlled type 2 diabetes. *Diabetes Research and Clinical Practice, 82*(1), 73–79. <https://doi.org/10.1016/j.diabres.2008.06.015> |

**Appendix 5. The List of Excluded Studies**

| **Year** | **Identification** | **Reference for Excluded Studies** | **Reasons** |
| --- | --- | --- | --- |
| 2016 | Databases and registers | Ajjan, R. A., Abougila, K., Bellary, S., Collier, A., Franke, B., Jude, E. B., Rayman, G., Robinson, A., & Singh, B. M. (2016). Sensor and software use for the glycaemic management of insulin-treated type 1 and type 2 diabetes patients. Diabetes & Vascular Disease Research, 13(3), 211–219. https://doi.org/10.1177/1479164115624680 | Intervention not relevant |
| 2019 | Databases and registers | Ajjan, R. A., Jackson, N., & Thomson, S. A. (2019). Reduction in HbA1c using professional flash glucose monitoring in insulin-treated type 2 diabetes patients managed in primary and secondary care settings: A pilot, multicentre, randomised controlled trial. Diabetes & Vascular Disease Research, 16(4), 385–395. https://doi.org/10.1177/1479164119827456 | Intervention not relevant |
| 2019 | Databases and registers | Furler, J., O’Neal, D. N., Speight, J., Manski-Nankervis, J. E., Thuraisingam, S., Holmes-Truscott, E., de la Rue, K. R., Ginnivan, L. E., Doyle, R. C., Khunti, K., Catchpool, M., Dalziel, K., Chiang, J. I., Blackberry, I., Audehm, R., Kennedy, M., Clark, M. J., Jenkins, A. J., Januszewski, A. S., ... Clarke, P. M. (2019). GP-OSMOTIC: An RCT to determine the effect of 3-monthly retrospective continuous glucose monitoring (rCGM) on 12-month HbA1c in adults with type 2 diabetes (T2D) in primary care. Diabetes, 68(Supplement_1), 102–LB. <https://doi.org/10.2337/db19-102-LBJ> | Intervention not relevant |
| 2014 | Other Methods | Blackberry, I. D., Furler, J. S., Ginnivan, L. E., Manski-Nankervis, J. A., Jenkins, A., Cohen, N., Best, J. D., Young, D., Liew, D., Ward, G., & O'Neal, D. N. (2014). An exploratory trial of basal and prandial insulin initiation and titration for type 2 diabetes in primary care with adjunct retrospective continuous glucose monitoring: INITIATION study. Diabetes Research and Clinical Practice, 106(2), 247–255. <https://doi.org/10.1016/j.diabres.2014.08.011> | Intervention not relevant |
| 2009 | Databases and registers | Cooke, D., Hurel, S. J., Casbard, A., Steed, L., Walker, S., Meredith, S., Nunn, A. J., Manca, A., Sculpher, M., Barnard, M., Kerr, D., Weaver, J. U., Ahlquist, J., & Newman, S. P. (2009). Randomized controlled trial to assess the impact of continuous glucose monitoring on HbA1c in insulin-treated diabetes (MITRE Study). Diabetic Medicine, 26(5), 540–547. <https://doi.org/10.1111/j.1464-5491.2009.02723.x> | Intervention not relevant |
| 2018 | Databases and registers | Ilany, J., Bhandari, H., Nabriski, D., Toledano, Y., Konvalina, N., & Cohen, O. (2018). Effect of prandial treatment timing adjustment, based on continuous glucose monitoring, in patients with type 2 diabetes uncontrolled with once-daily basal insulin: A randomized, phase IV study. Diabetes, Obesity and Metabolism, 20(5), 1186–1192. https://doi.org/10.1111/dom.13214 | Intervention not relevant |
| **Year** | **Identification** | **Reference for Excluded Studies** | **Reasons** |
| 2016 | Databases and registers | Sato, J., Kanazawa, A., Ikeda, F., Shigihara, N., Kawaguchi, M., Komiya, K., Uchida, T., Ogihara, T., Mita, T., Shimizu, T., Fujitani, Y., & Watada, H. (2016). Effect of treatment guidance using a retrospective continuous glucose monitoring system on glycaemic control in outpatients with type 2 diabetes mellitus: A randomized controlled trial. Journal of International Medical Research, 44(1), 109–121. <https://doi.org/10.1177/0300060515600190> | Intervention not relevant |
| 2018 | Databases and registers | Yeoh, E., Lim, B. K., Fun, S., Tong, J., Yeoh, L. Y., Sum, C. F., Subramaniam, T., & Lim, S. C. (2018). Efficacy of self-monitoring of blood glucose versus retrospective continuous glucose monitoring in improving glycaemic control in diabetic kidney disease patients. Nephrology (Carlton), 23(3), 264–268. <https://doi.org/10.1111/nep.12978> | Intervention not relevant |
| 2023 | Databases and registers | Lind, N., Christensen, M. B., & Nørgaard, K. (2023). CGM-derived metrics and HbA1c in adults with type 2 diabetes (T2D) on different insulin treatment regimens not meeting glycemic targets. Diabetes, 72(Supplement_1), 1817–PUB. <https://doi.org/10.2337/db23-1817-PUB> | Intervention not relevant |
| 2024 | Databases and registers | Idrees, T., Castro-Revoredo, I. A., Oh, H. D., Johnson, T. M., Peng, L., & Umpierrez, G. E. (2024). Continuous glucose monitoring-guided insulin administration in long-term care facilities: A randomized clinical trial. Diabetes Technology & Therapeutics, 25(5), 884–888. <https://doi.org/10.xxxx/xxxxx> | Intervention not relevant |
| 2010 | Databases and registers | Chen, X. M., Zhang, Y., Shen, X. P., Huang, Q., Ma, H., Huang, Y. L., Zhang, W. Q., & Wu, H. J. (2010). Correlation between glucose fluctuations and carotid intima-media thickness in type 2 diabetes. Diabetes Research and Clinical Practice, 90(1), 95–99. <https://doi.org/10.1016/j.diabres.2010.05.004> | Intervention not relevant |
| 2020 | Databases and registers | Chiang, J. I., Manski-Nankervis, J. A., Thuraisingam, S., Jenkins, A., O'Neal, D., Mair, F. S., Jani, B. D., Nicholl, B. I., & Furler, J. (2020). Multimorbidity, glycaemic variability and time in target range in people with type 2 diabetes: A baseline analysis of the GP-OSMOTIC trial. Diabetes Research and Clinical Practice, 169, 108451. <https://doi.org/10.1016/j.diabres.2020.108451> | Intervention not relevant |
| 2017 | Databases and registers | Costantino, S., Paneni, F., Battista, R., Castello, L., Capretti, G., Chiandotto, S., Tanese, L., Russo, G., Pitocco, D., Lanza, G. A., Volpe, M., Lüscher, T. F., & Cosentino, F. (2017). Impact of glycemic variability on chromatin remodeling, oxidative stress, and endothelial dysfunction in patients with type 2 diabetes and with target HbA1c levels. Diabetes, 66(9), 2472–2482. <https://doi.org/10.2337/db17-0294> | Intervention not relevant |
| **Year** | **Identification** | **Reference for Excluded Studies** | **Reasons** |
| 2021 | Databases and registers | Oser, T. K., Litchman, M. L., Allen, N. A., Kwan, B. M., Fisher, L., Jortberg, B. T., Polonsky, W. H., & Oser, S. M. (2021). Personal continuous glucose monitoring use among adults with type 2 diabetes: Clinical efficacy and economic impacts. Current Diabetes Reports, 21, 49. <https://doi.org/10.1007/s11892-021-01408-1> | Intervention not relevant |
| 2024 | Databases and registers | Kim, J. Y., Jin, S. M., Sim, K. H., Kim, B. Y., Cho, J. H., Moon, J. S., Lim, S., Kang, E. S., Park, C. Y., Kim, S. G., & Kim, J. H. (2024). Continuous glucose monitoring with structured education in adults with type 2 diabetes managed by multiple daily insulin injections: A multicentre randomised controlled trial. Diabetologia, 67(7), 1223–1234. <https://doi.org/10.1007/s00125-024-06152-1> | Intervention not relevant |
| 2008 | Other Methods | Allen, N. A., Fain, J. A., Braun, B., & Chipkin, S. R. (2008). Continuous glucose monitoring counseling improves physical activity behaviors of individuals with type 2 diabetes: A randomized clinical trial. Diabetes Research and Clinical Practice, 80(3), 371–379. <https://doi.org/10.1016/j.diabres.2008.01.006> | Duration of study not relevant |
| 2021 | Databases and registers | Rivera-Ávila, D. A., Esquivel-Lu, A. I., Salazar-Lozano, C. R., Jones, K., & Doubova, S. V. (2021). The effects of professional continuous glucose monitoring as an adjuvant educational tool for improving glycemic control in patients with type 2 diabetes. BMC Endocrine Disorders, 21(1), 79. https://doi.org/10.1186/s12902-021-00742-5 | Study design not relevant |
| 2007 | Databases and registers | Zick, R., Petersen, B., Richter, M., Haug, C., & SAFIR Study Group. (2007). Comparison of continuous blood glucose measurement with conventional documentation of hypoglycemia in patients with type 2 diabetes on multiple daily insulin injection therapy. Diabetes Technology & Therapeutics, 9(6), 483–492. <https://doi.org/10.1089/dia.2007.0230> | Study design not relevant |
| 2013 | Databases and registers | Oliveira, A. O. T. de., Bartholomew, K., Lavin-Tompkins, J., & Sperl-Hillen, J. (2013). Use of continuous glucose monitoring as an educational tool in the primary care setting. Diabetes Spectrum, 26(2), 120–123. <https://doi.org/10.2337/diaspect.26.2.120> | Study design not relevant |
| 2010 | Databases and registers | Fritschi, C., Quinn, L., Penckofer, S., & Surdyk, P. M. (2010). Continuous glucose monitoring: The experience of women with type 2 diabetes. *The Diabetes Educator, 36*(2), 250–257. <https://doi.org/10.1177/0145721709355835> | Study design not relevant |
| **Year** | **Identification** | **Reference for Excluded Studies** | **Reasons** |
| 2019 | Other Methods | Abe, H., Shikuma, J., Suwanai, H., Sano, K., Okumura, T., Kan, K., Takahashi, T., Miwa, T., Suzuki, R., & Odawara, M. (2019). Assessing hypoglycemia frequency using flash glucose monitoring in older Japanese patients with type 2 diabetes receiving oral hypoglycemic agents. Geriatrics & Gerontology International, 19(10), 1030–1035. <https://doi.org/10.1111/ggi.13765> | Comparator not relevant |
| 2019 | Databases and registers | Alawadi, F., Rashid, F., Bashier, A., Abdelgadir, E., Al Saeed, M., Abuelkheir, S., Khalifa, A., Al Sayyah, F., Bachet, F., Elsayed, M., Abdallah, K., & Hassanein, M. (2019). The use of FreeStyle Libre continuous glucose monitoring (FSL-CGM) to monitor the impact of Ramadan fasting on glycemic changes and kidney function in high-risk patients with diabetes and chronic kidney disease stage 3 under optimal diabetes care. Diabetes Research and Clinical Practice, 151, 305–312. <https://doi.org/10.1016/j.diabres.2019.03.015> | Comparator not relevant |
| 2018 | Databases and registers | Lu, J., Ma, X., Zhou, J., Zhang, L., Mo, Y., Ying, L., Lu, W., Zhu, W., Bao, Y., Vigersky, R. A., & Jia, W. (2018). Association of time in range, as assessed by continuous glucose monitoring, with diabetic retinopathy in type 2 diabetes. Diabetes Care, 41(11), 2370–2376. <https://doi.org/10.2337/dc18-1131> | Comparator not relevant |
| 2022 | Databases and registers | Clark, C. N., Hart, B. B., McNeil, C. K., Duerr, J. M., & Weller, G. B. (2022). Improved time in range during 28 days of meal delivery for people with type 2 diabetes. Diabetes Spectrum, 35(3), 358–366. <https://doi.org/10.2337/ds21-0093> | Comparator not relevant |
| 2022 | Databases and registers | Kazda, C., Chien, J. Y., Zhang, Q., Chigutsa, E., Landschulz, W. H., Wullenweber, P. K., Haupt, A., Frias, J., & Forst, T. (2022). Glycemic control with once-weekly basal insulin Fc (BIF) in persons with type 2 diabetes mellitus (T2DM) using continuous glucose monitoring (CGM) in a phase 2 study. Diabetologie und Stoffwechsel, 17(S 01), S27. <https://doi.org/10.1055/s-0042-1746283> | Comparator not relevant |
| 2018 | Databases and registers | Shrivastav, M., Gibson, W., Jr., Shrivastav, R., Elzea, K., Khambatta, C., Sonawane, R., Sierra, J. A., & Vigersky, R. (2018). Type 2 diabetes management in primary care: The role of retrospective, professional continuous glucose monitoring. Diabetes Spectrum, 31(3), 279–287. <https://doi.org/10.2337/ds17-0024> | Review paper |
| 2021 | Databases and registers | Gavin, J. R., & Bailey, C. J. (2021). Real-world studies support use of continuous glucose monitoring in type 1 and type 2 diabetes independently of treatment regimen. Diabetes Technology & Therapeutics, 23(S3), S19–S27. <https://doi.org/10.1089/dia.2021.0211> | Review paper |
| **Year** | **Identification** | **Reference for Excluded Studies** | **Reasons** |
| 2020 | Databases and registers | Zheng, M., Luo, Y., Lin, W., Khoja, A., He, Q., Yang, S., Zhao, X., & Hu, P. (2020). Comparing effects of continuous glucose monitoring systems (CGMs) and self-monitoring of blood glucose (SMBG) amongst adults with type 2 diabetes mellitus: A systematic review protocol. Systematic Reviews, 9(1), 120. <https://doi.org/10.1186/s13643-020-01386-7> | Review paper |
| 2023 | Databases and registers | DePietro, R., & Cronk, N. J. (2023). In patients with type 2 diabetes not taking insulin, does continuous glucose monitoring improve glucose control? *Evidence-Based Practice, 26*(7), 16–17. <https://doi.org/10.1097/EBP.0000000000001860> | Review paper |
| 2017 | Databases and registers | Chan, C. L. (2017). Use of continuous glucose monitoring in youth-onset type 2 diabetes. Current Diabetes Reports, 17(9), 66. <https://doi.org/10.1007/s11892-017-0905-0> | Population not relevant |
| 2024 | Databases and registers | Lind, N., Christensen, M. B., Hansen, D. L., & Nørgaard, K. (2024). Comparing continuous glucose monitoring and blood glucose monitoring in adults with inadequately controlled, insulin-treated type 2 diabetes (Steno2tech study): A 12-month, single-center, randomized controlled trial. Diabetes Care, 47(5), 881–889. <https://doi.org/10.2337/dc23-2194> | Population not relevant |
| 2000 | Databases and registers | Boland, E. A., & Tamborlane, W. V. (2000). Continuous glucose monitoring in youth with type 2 diabetes: Overcoming barriers to successful treatment. Diabetes Technology & Therapeutics, 2(Suppl 1), S53–S59. <https://doi.org/10.1089/15209150050214131> | Population not relevant |
| 2015 | Databases and registers | New, J. P., Ajjan, R., Pfeiffer, A. F., & Freckmann, G. (2015). Continuous glucose monitoring in people with diabetes: The randomized controlled Glucose Level Awareness in Diabetes Study (GLADIS). Diabetic Medicine, 32(5), 609–617. <https://doi.org/10.1111/dme.12713> | Population not relevant |
| 2017 | Databases and registers | Ruedy, K. J., Parkin, C. G., Riddlesworth, T. D., Graham, C., & DIAMOND Study Group. (2017). Continuous glucose monitoring in older adults with type 1 and type 2 diabetes using multiple daily injections of insulin: Results from the DIAMOND trial. Journal of Diabetes Science and Technology, 11(6), 1138–1146. <https://doi.org/10.1177/1932296817704445> | Population not relevant |
| 2024 | Databases and registers | Lever, C. S., Williman, J. A., Boucsein, A., Watson, A., Sampson, R. S., Sergel-Stringer, O. T., Keesing, C., Chepulis, L., Wheeler, B. J., de Bock, M. I., & Paul, R. G. (2024). Real time continuous glucose monitoring in high-risk people with insulin-requiring type 2 diabetes: A randomised controlled trial. Diabetic Medicine, 41(8), e15348. <https://doi.org/10.1111/dme.15348> | Population not relevant |
| **Year** | **Identification** | **Reference for Excluded Studies** | **Reasons** |
| 2022 | Databases and registers | Isaacson, B., Kaufusi, S., Sorensen, J., Joy, E., Jones, C., Ingram, V., Mark, N., Phillips, M., & Briesacher, M. (2022). Demonstrating the clinical impact of continuous glucose monitoring within an integrated healthcare delivery system. Journal of Diabetes Science and Technology, 16(2), 383–389. <https://doi.org/10.1177/1932296820955228> | Population not relevant |
| 2017 | Databases and registers | Aleppo, G., Ruedy, K. J., Riddlesworth, T. D., Kruger, D. F., Peters, A. L., Hirsch, I., Bergenstal, R. M., Toschi, E., Ahmann, A. J., Shah, V. N., Rickels, M. R., Bode, B. W., Philis-Tsimikas, A., Pop-Busui, R., Rodriguez, H., Eyth, E., Bhargava, A., Kollman, C., & Beck, R. W. (2017). REPLACE-BG: A randomized trial comparing continuous glucose monitoring with and without routine blood glucose monitoring in adults with well-controlled type 1 diabetes. *Diabetes Care, 40*(4), 538–545. <https://doi.org/10.2337/dc16-248> | Population not relevant |
| 2017 | Databases and registers | Arguello, V., & Freeby, M. (2017). Continuous glucose monitoring in patients with type 2 diabetes receiving insulin injections: Does this mean continuous glucose monitoring for everyone? Annals of Internal Medicine, 167(6), 436–437. <https://doi.org/10.7326/M17-212> | Letter to editor |
| 2018 | Databases and registers | Beck, R. W., & Riddlesworth, T. D. (2018). Continuous glucose monitoring versus usual care in patients with type 2 diabetes receiving multiple daily insulin injections. Annals of Internal Medicine, 168(7), 526–527. <https://doi.org/10.7326/L17-0706> | Letter to editor |
| 2021 | Databases and registers | Cowart, K., & Carris, N. W. (2021). Continuous glucose monitoring and glycemic control in patients with type 2 diabetes treated with basal insulin. *JAMA, 326*(13), 1329–1330. <https://doi.org/10.1001/jama.2021.13472> | Letter to editor |
| 2022 | Databases and registers | Bao, S., Bailey, R., Calhoun, P., & Beck, R. W. (2022). Effectiveness of continuous glucose monitoring in older adults with type 2 diabetes treated with basal insulin. *Diabetes Technology & Therapeutics, 24*(5), 299–306. <https://doi.org/10.1089/dia.2021.0494> | Outcome not relevant |
| 2021 | Databases and registers | Aleppo, G., Beck, R. W., Bailey, R., Ruedy, K. J., Calhoun, P., Peters, A. L., Pop-Busui, R., Philis-Tsimikas, A., Bao, S., Umpierrez, G., Davis, G., Kruger, D., Bhargava, A., Young, L., Buse, J. B., McGill, J. B., Martens, T., Nguyen, Q. T., Orozco, I., Biggs, W., Lucas, K. J., Polonsky, W. H., Price, D., & Bergenstal, R. M.; MOBILE Study Group. (2021). The effect of discontinuing continuous glucose monitoring in adults with type 2 diabetes treated with basal insulin. *Diabetes Care, 44*(12), 2729–2737. <https://doi.org/10.2337/dc21-1304> | Same study population |
| **Year** | **Identification** | **Reference for Excluded Studies** | **Reasons** |
| 2013 | Databases and registers | Tildesley, H. D., Wright, A. M., Chan, J. H., Mazanderani, A. B., Ross, S. A., Tildesley, H. G., et al. (2013). A comparison of internet monitoring with continuous glucose monitoring in insulin-requiring type 2 diabetes mellitus. *Canadian Journal of Diabetes, 37*(5), 305–308. | Same study population |
| 2011 | Databases and registers | Ehrhardt, N. M., Chellappa, M., Walker, M. S., Fonda, S. J., & Vigersky, R. A. (2011). The effect of real-time continuous glucose monitoring on glycemic control in patients with type 2 diabetes mellitus. *Journal of Diabetes Science and Technology, 5*(3), 668–675. | Same study  population |

**Appendix 6. Risk of Bias Summary**

**
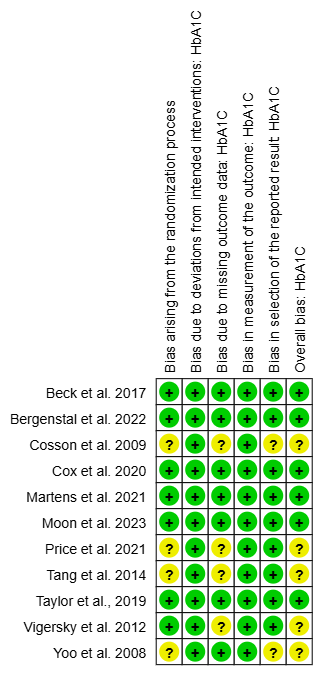
**

**Appendix 7: Forest Plot of the Effect of rtCGM on HbA_1C_ Compared with SMBG**


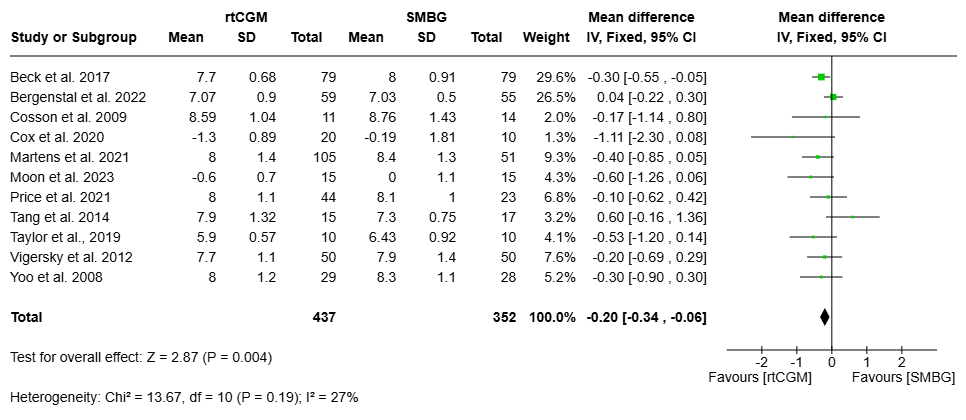


**Appendix 8: Forest Plot of the Effect of rtCGM on Glucose Metrics (TIR, TAR, TBR) Compared with SMBG**

1. **The effect of rtCGM on TIR**


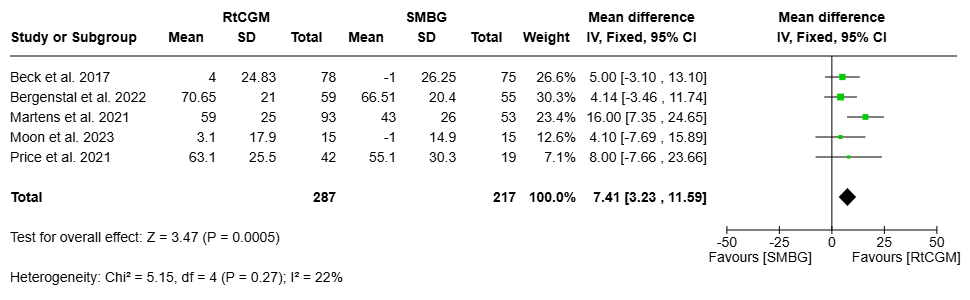


1. **The effect of rtCGM on TAR**


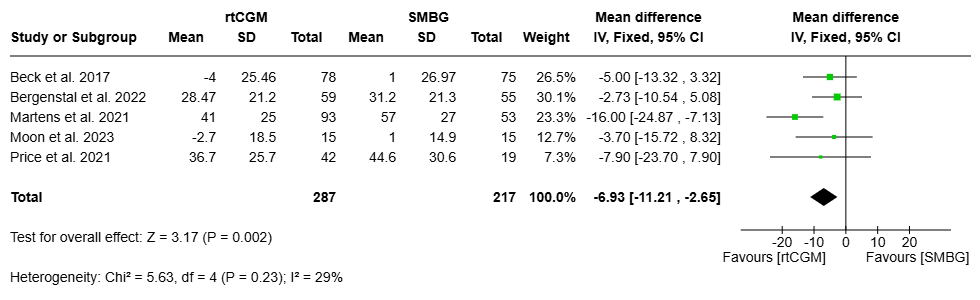


1. **The Effect of rtCGM on TBR**


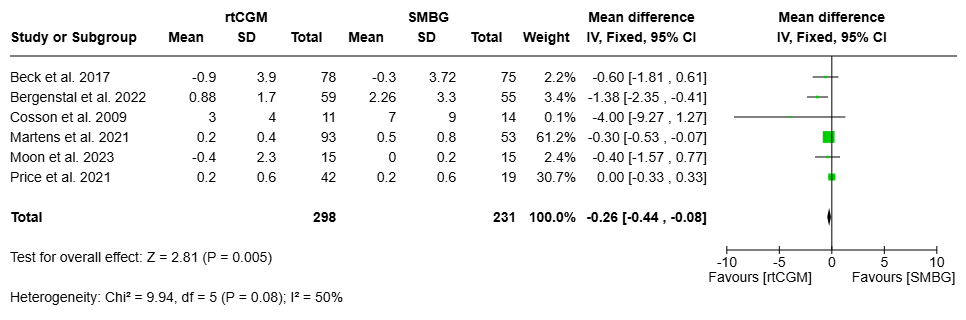


1. **The effect of rtCGM on glucose variability**


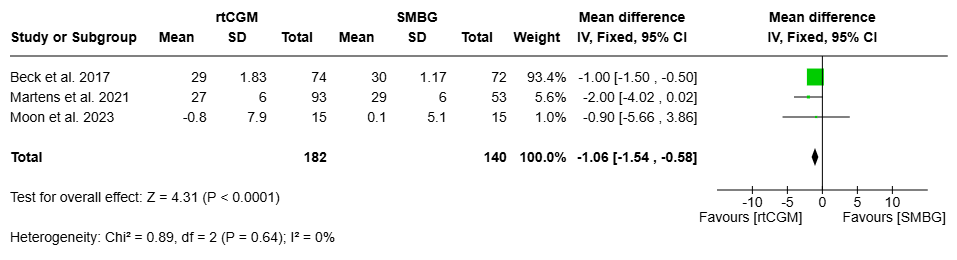


**Appendix 9: Forest Plot of the Effect of rtCGM on Cardiometabolic Parameters (Body Weight and BMI) Compared with SMBG**

1. **The Effect of rtCGM on Body Weight**

**
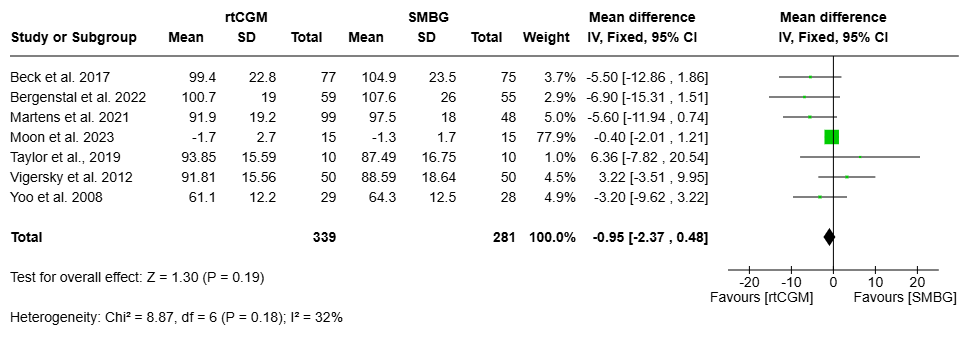
**

1. **The Effect of rtCGM on BMI**

**
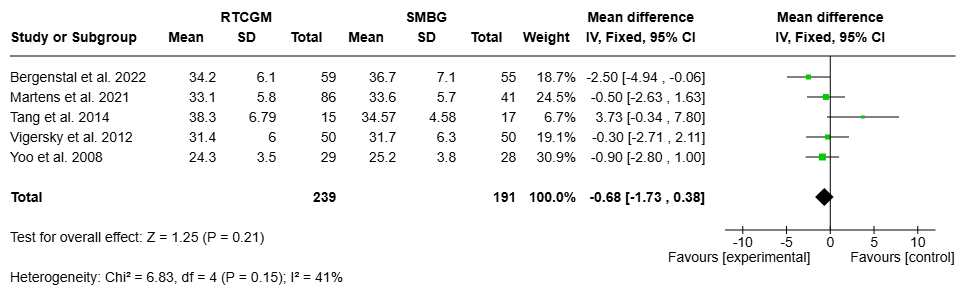
**

**Appendix 10: Forest Plot of the Effect of rtCGM on Cardiometabolic Parameters (LDL, HDL and TG) Compared with SMBG**

**
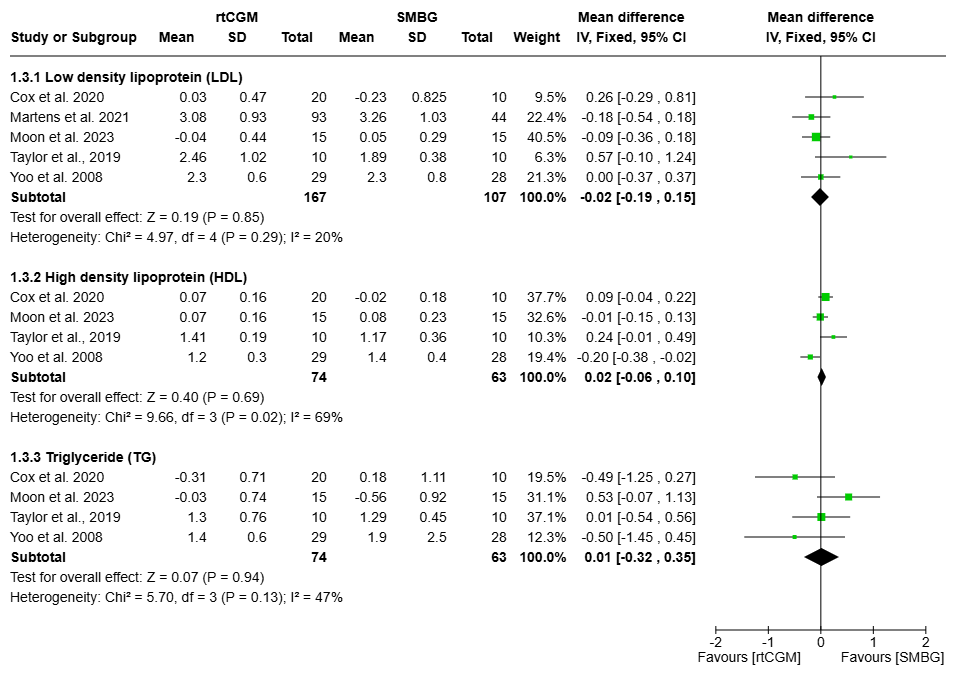
**

**Appendix 11: Forest Plot of the Effect of rtCGM on Cardiometabolic Parameters (SBP and DBP) Compared with SMBG**

**
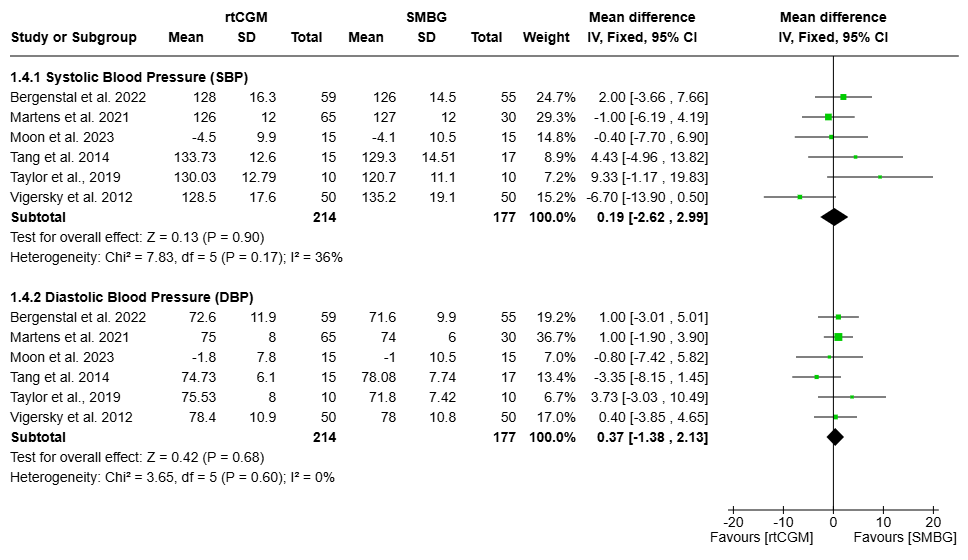
**

**Appendix 12: Forest Plot of the Effect of rtCGM on Self-Care Behaviour Compared with SMBG**

1. **Change in Total Calorie Intake**

**
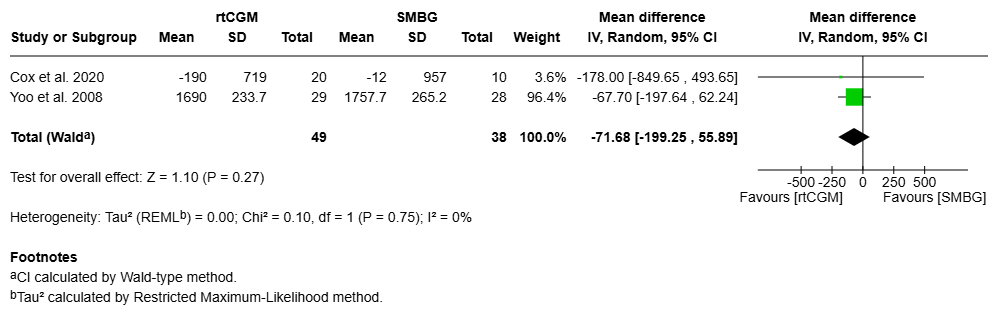
**

**2. Change in Carbohydrate Intake**

**
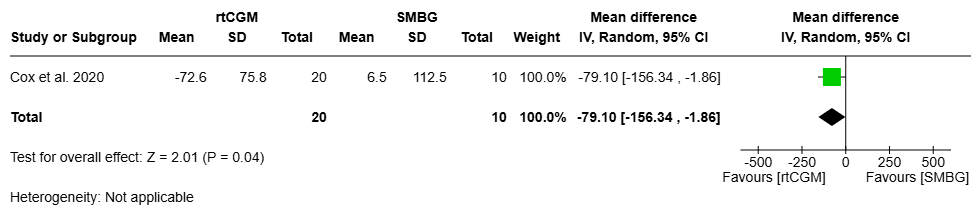
**

**3. Change in Exercise Time**

**
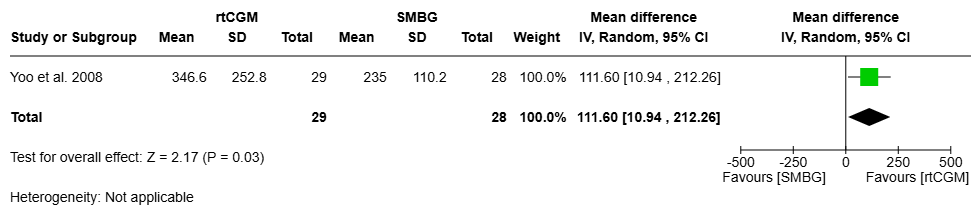
**

**4. Change in Glucose Monitoring Frequency**

**
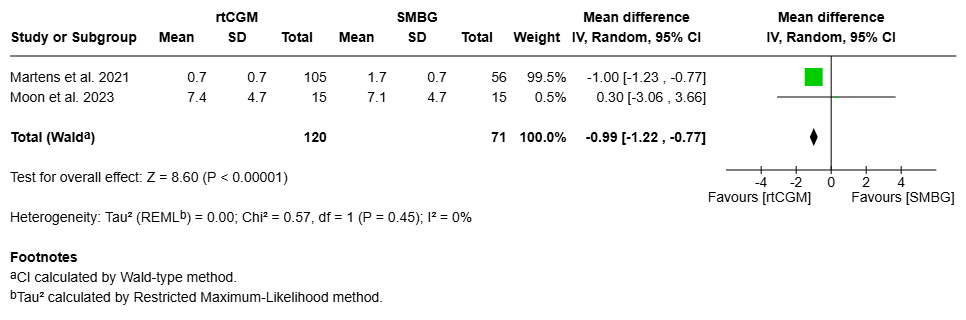
**

1. **Change in Diabetes Knowledge**

**
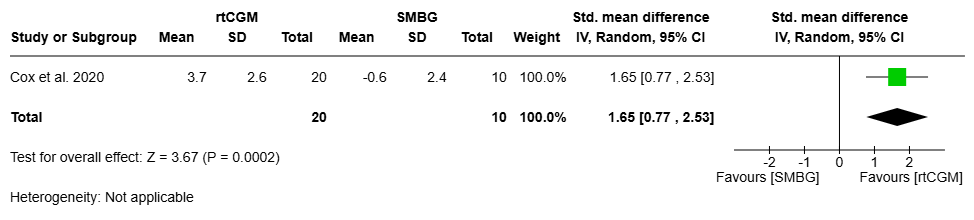
**

1. **Readiness for Diabetes Self- Management**


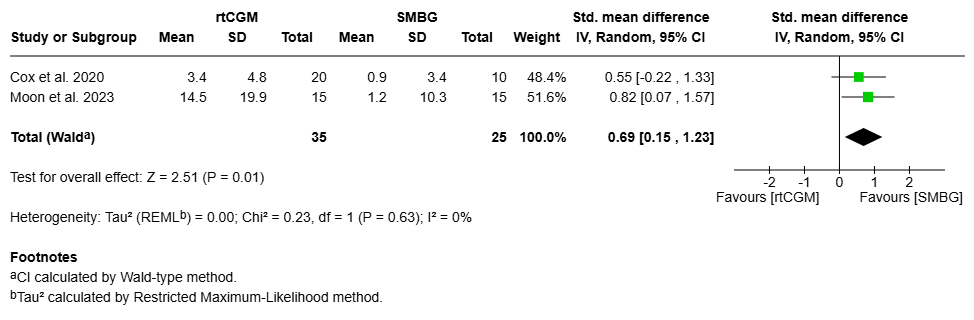


**Appendix 13. Forest Plot of the Effect of rtCGM on Satisfaction and Quality of Life Compared with SMBG**

**
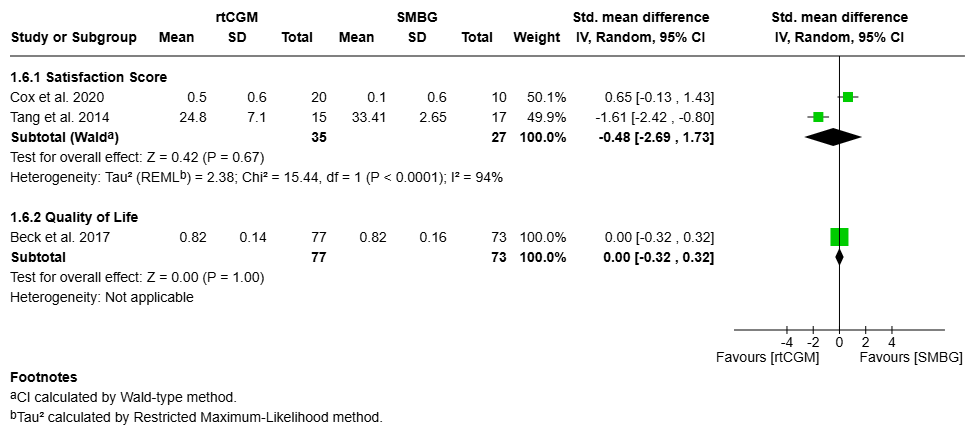
**

**Appendix 14: Risk of Adverse Events of rtCGM Compared with SMBG**

**
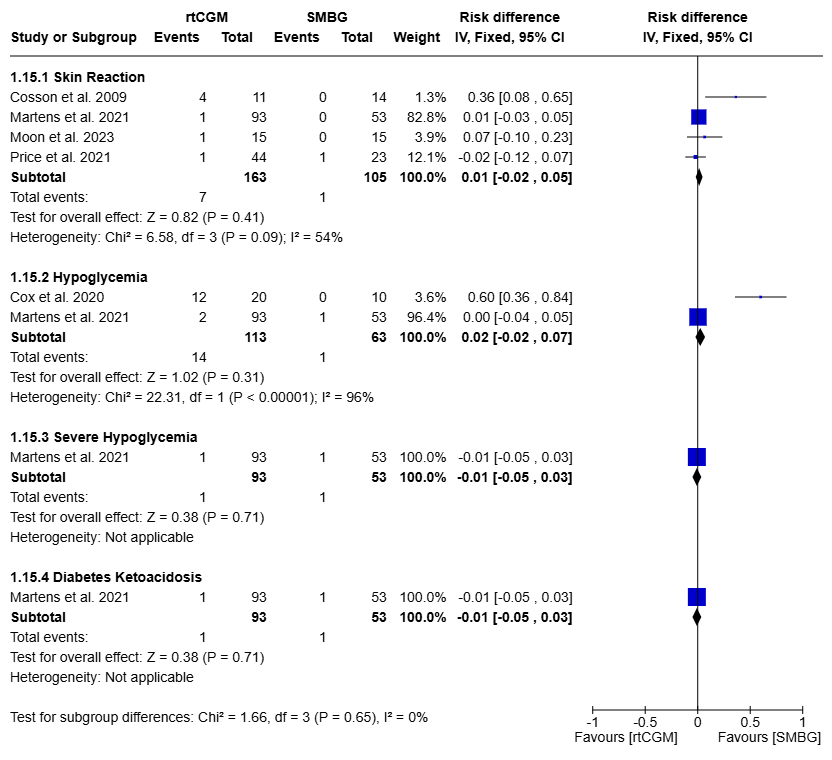
**

**Appendix 15: Funnel Plot for Change in HbA_1c_ Levels Comparing rtCGM with SMBG**


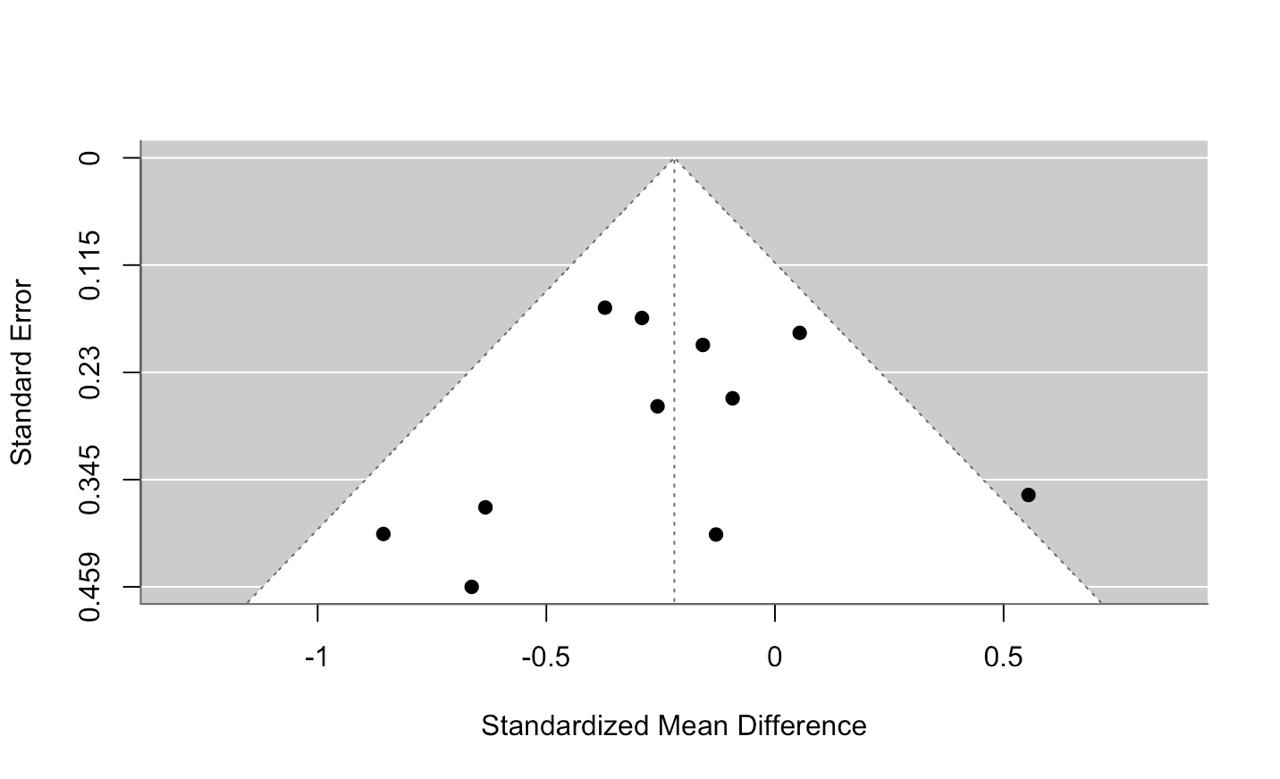


**Appendix 16: GRADE Summary of Findings**

| **Certainty assessment** | | | | | | | **№ of patients** | | **Effect** | **Certainty** | **Importance** |
| --- | --- | --- | --- | --- | --- | --- | --- | --- | --- | --- | --- |
| **№ of studies** | **Study design** | **Risk of bias** | **Inconsistency** | **Indirectness** | **Imprecision** | **Other considerations** | **rt CGM** | **SMBG** | **Relative (95% CI)** |  |  |
| **Glycated Hemoglobin (HbA_1C_)** | | | | | | | | | | | |
| 11 | RCT | not serious | not  serious | not serious | not serious | none | 437 | 352 | MD -0.20%  (-0.34, -0.06) | ⨁⨁⨁◯ Moderate | IMPORTANT |
| **Time in Range (TIR)** | | | | | | | | | | | |
| 5 | RCT | not serious | not  serious | not serious | not serious | none | 287 | 217 | MD 7.41 % (3.23, 11.59) | ⨁⨁⨁◯ Moderate | IMPORTANT |
| **Time Above Range (TAR)** | | | | | | | | | | | |
| 5 | RCT | not serious | not  serious | not serious | not serious | none | 287 | 217 | MD -6.93%  (-11.21, -2.65) | ⨁⨁⨁◯ Moderate | IMPORTANT |
| **Time Below Range (TBR)** | | | | | | | | | | | |
| 6 | RCT | not serious | serious | not serious | not serious | none | 298 | 231 | MD -0.26% (-0.44, -0.08) | ⨁⨁◯◯ Low | IMPORTANT |
| **Glucose Variability** | | | | | | | | | | | |
| 3 | RCT | not serious | not  serious | not serious | not serious | none | 182 | 140 | MD-1.06%  (-1.54, -0.58) | ⨁⨁⨁◯ Moderate | IMPORTANT |
